# Genetic associations of prostate cancer in China: a systematic review

**DOI:** 10.1186/s12885-025-13830-9

**Published:** 2025-04-03

**Authors:** Yimin Pang, Junjun Li, Hao Hu, Carolina Oi Lam Ung

**Affiliations:** 1 State Key Laboratory of Quality Research in Chinese Medicine, Institute of Chinese Medical Sciences, University of Macau, Macao SAR, China; 2 Centre for Pharmaceutical Regulatory Sciences, University of Macau, Macao SAR, China; 3 Department of Public Health and Medicinal Administration, Faculty of Health Sciences, University of Macau, Macao SAR, China

**Keywords:** Prostate cancer, Genetic association, Systematic review

## Abstract

**Objectives:**

In recent years, there has been a notable increase in the incidence and mortality rates of prostate cancer (PCa) in China, highlighting it as a significant public health issue. This study aimed to investigate the genetic association of PCa in China to better inform national disease management and medical resource allocation.

**Methods:**

A systematic literature review was conducted using 5 English databases (Web of Science, PubMed, Embase, Cochrane, Scopus) and 1 Chinese database (CNKI) to identify articles published from database inception to October 8, 2022, which reported the genetic associations of PCa in China.

**Results:**

Of the 11,195 articles retrieved, 41 were included in the review. A total of 116 different polymorphisms (including single nucleotide polymorphisms, deletions, insertions, and repeat lengths) in 58 genes were studied in Chinese populations. Among these, 37 out of 51 polymorphisms in 28 candidate genes such as BIRC5, C2orf43, COX-2, CYR61 (IGFBP10), DNMT1, DNMT3B, EXO1, FOXP4, and 7 unmapped SNPs were found to have either a positive or negative effect on PCa risk. However, 18 variants in 5 genes remain controversial across different studies. Additionally, 23 SNPs in 16 genes were reported to be associated with disease stage, Gleason score, PSA levels, PCa risk, and clinicopathological characteristics of PCa in China.

**Conclusion:**

In Chinese populations, PCa risk and clinical features may result from individual genes, gene-gene interactions, and gene-environment interactions. These findings provide important insights into the relationship between genetic susceptibility and PCa risk in Chinese men.

**Supplementary Information:**

The online version contains supplementary material available at 10.1186/s12885-025-13830-9.

## Introduction

Prostate cancer (PCa) is emerging as a significant public health concern, significantly impacting the health of men in China. Epidemiological data from 2020 indicated that the incidence of PCa in China was much lower compared to European and African countries. However, new cases and deaths from PCa in China accounted for 8.2% and 13.6% of the global total, respectively [1]. Furthermore, several studies projected that by 2030, the incidence of PCa in China will rise from the seventh most common malignancy to the third, with a percentage change (2015–2030) of 517% [1, 2]. Given the substantial implications for public health and the well-being of Chinese males, the rapid increase in incidence rates, and the vast number of affected individuals, there is a pressing need for a deeper understanding of the etiology of PCa in China.

Currently, the etiology of PCa remains largely elusive, with genetic syndromes, familial predisposition, and race identified as established risk factors [3]. Genetic factors, in particular, were recognized as one of the few established risk factors for PCa. Quantitative estimates from twin studies suggested that approximately 42% of the variability in PCa risk might be attributed to genetic factors, surpassing other common malignancies [4]. This underscores the potentially significant role of genetic predisposition in individual susceptibility to PCa. Additionally, previous research indicated that PCa incidence among Chinese immigrants in the United States was only half that of Caucasian individuals born in the United States, suggesting differential genetic susceptibility to the clinical manifestation of PCa development in Asian populations compared to Caucasians [5].

To identify genetic alterations associated with the risk of PCa, over 40 genome-wide association studies (GWAS) have been conducted, revealing more than 200 PCa susceptibility loci [6–8]. However, the majority of these loci were found from studies focusing on European populations. This suggested that the current research about the important risk alleles in Asian populations might be potentially underreported [9].Current GWAS studies showed that Asian populations exhibited different frequencies of risk alleles at certain risk loci compared to European, African, and Hispanic populations [10]. For instance, GWAS conducted in Chinese populations identified two independent susceptibility loci for PCa (9q31.2 rs817826 and 19q13.4 rs103294), highlighting distinct differences in both the risk and genetic background of PCa among Chinese males compared to other ethnicities [11].Importantly, while GWAS reports have begun to elucidate the combined effects of risk loci, many of the biological implications of PCa susceptibility loci remain unclear.

Considering that the current research indicates that the incidence of PCa in China is significantly lower than in Western populations, whether there are unique epidemiological characteristics that may be associated with distinct genetic backgrounds is worth further investigation [12]. However, despite recognized racial disparities, research is scarce about PCa in the Chinese population. Most published data from genomic research and clinical trials focused on Western populations. The lack of data represents a major limitation in developing specific diagnostic and therapeutic approaches tailored to Asian males. This study aims to address this research gap through a systematic review, by identifying potential candidate genes and their biological significance, as well as reporting on the combined effects of risk variants, interactions with the environment, and their impact on clinical features. These genetic associations identified in this study could be further validated in future studies using large samples from diverse racial groups and used to inform continuous research about the identification of multiple genes involved in different pathways of PCa susceptibility.

## Methods

### Search strategy and selection criteria

This systematic review was conducted and reported in adherence to the PRISMA guidelines [13]. We systematically searched five English databases (Web of Science, PubMed, Embase, Scopus, Cochrane Central) and one Chinese database (CNKI) to gather relevant literature on the genetic factors of PCa in China. By including CNKI, the prominent Chinese electronic database, a comprehensive search of relevant literature and coverage of relevant Chinese-language studies was ensured. Additionally, we conducted grey literature searches. The search spanned from January 1, 2012, to October 1, 2022. Detailed search strategies are provided in the Supplementary Information.

Two rounds of screening were conducted for inclusion evaluation. In the initial round, studies were excluded based on title and abstract if they fell into the following categories: (1) case reports, letters, comments, systematic reviews/meta-analyses, reviews, guidelines, conference abstracts, early access articles, editorial materials, corrections, notes, retracted publications, or protocols; (2) studies not related to PCa or its risk factors; (3) studies involving animal or cellular experiments; (4) studies with populations outside of China; (5) non-case-control study or studies involving less than 100 PCa patients; (6) Data preceding 2012. The full-text evaluation was conducted in the second round to identify publications reporting on genetic risk factors in Chinese PCa patients, including candidate gene association studies (CGAS) and genome-wide association studies (GWAS). Furthermore, the references of included articles were screened to identify additional eligible literature. Two authors independently assessed titles, abstracts, and full texts throughout the screening process to ensure consistency and objectivity in inclusion decisions. Any discrepancies between the authors' assessments were resolved through discussion, and all references were managed using EndNote X9 software.

### Data analysis

The second round of screening involved a thorough review of full texts to identify publications that reported genetic risk factors in Chinese patients with PCa. Additionally, the references of included articles were scrutinized to uncover further eligible literature. Two authors independently evaluated titles, abstracts, and full texts of articles to ensure consistency and objectivity in the inclusion process. Any disparities between the authors' assessments were resolved through discussion, and all references were managed using EndNote X9 software.

Data analysis involved categorizing the included studies based on the gene, polymorphisms, and effect allele associated with PCa. Information extracted from each article included the first author, year of publication, study type, ethnicity, mean age of participants, sample source, sample size, genotyping method, gene information, odds ratios, or other relevant effects. Through the extraction and analysis of gene-related risk factors, a summary of genes implicated in PCa recurrence and progression to metastatic disease was compiled.

### Quality assessment

To assess the risk of bias in the included case-control studies, the Newcastle-Ottawa Scale tool [14] was used. All included articles were independently reviewed and evaluated by 2 authors. Negotiated and resolved differences between the 2 authors. Excel 2016 is used to facilitate data extraction and documentation.

## Results

### Characteristics of the included studies

**Fig. 1:**
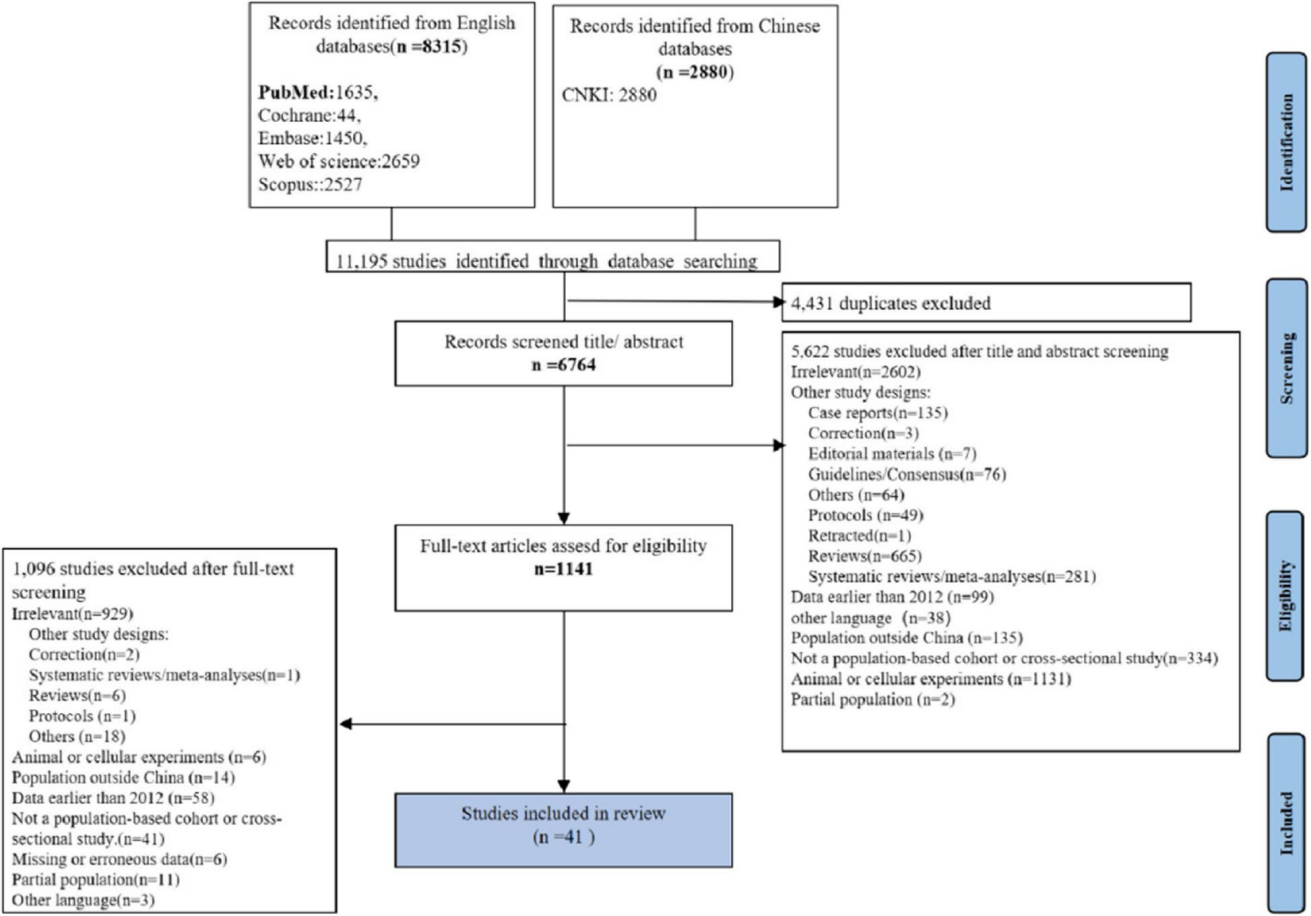
PRISMA flowchart of study selection

We initially retrieved 11,195 articles, of which 9970 were excluded during the removal of duplication, and title and abstract screening. Among the remaining 1,141 articles that might be relevant, 41 full-text case-control studies were included (Fig. 1). The general information of the included publications and the results of the study quality assessment are summarized in Table 1.

**Table 1:** Summary of reported basic information from all included studies and estimated quality score for each study

Author Years)	Study type	Ethnicity	Size (case/control)	Mean age (case/control)	Source*	Genotyping method*	Gene	Polymorphism*	**Quality Score**
Cao *et al* (2018) [15]	CGAS	Han	1015/1032	NA	Fudan University Shanghai Cancer Center(FUSCC)	TaqMan	PCA3	rs544190	6
Luo *et al* (2018) [16]	CGAS	Yi	122/135	NA	HB	Sanger	SRD5A2	rs523349 rs9282858 TA repeat site	7
Chen *et al* (2017) [17]	CGAS	Han	268/298	58/62	Institution	HRM-PCR	KLK3	rs1058205	6
Chen *et al* (2016) [18]	CGAS	Han	439/524	69.85±8.66/69.39±8.74	HB	PCR-RFLP	IL-4 IL-6 IL-8 NFKBIA	IL-4-rs2243250 IL-6-rs10499563 IL-8-rs4073NFKBIA-rs2233406, rs3138053	7
Chen *et al* (2013) [19]	CGAS	Han	665/710	NA	HB	TaqMan	BIRC5	SNP rs9904341	6
Cui *et al* (2015) [20]	CGAS	NA	543/753	69.90 ± 8.43/69.38 ± 8.76	HB	PCR-RFLP	NFKB1,NFKBIA,COX-1,COX-2	NFKB1-94ins/del Ins/Ins Ins/Del Del/Del NFKBIA-826C/T COX-2(−1195G>A) COX-1(50C>T) PPARG Pro12Ala	7
Han *et al* (2015) [21]	CGAS	Han	936/936	62.0±6.89/61.4±7.11	HB	PCR-RFLP	NFKB1 and NFKBIA	NFKB1 Ins/ins Ins/Del Del/del NFKBIA 3'UTR NFKBIA-826CT NFKBIA-881AG	5
He *et al* (2014) [22]	CGAS	Han	155/155	68.13 ± 6.22/68.56 ± 6.73	HB	Sequenom MassARRAY	DNMT1, DNMT3B	DNMT1 (rs16999593, rs2228611), DNMT3B (rs2424908	7
Huang *et al* (2016) [23]	CGAS	Han	236/256	NA	HB	PCR-RFLP	IL6 promoter	rs1800796	7
Li *et al* (2013) [24]	CGAS	Han, Uygur	1004/1051	69.0±8.16/69.0±8.96	Fudan University Shanghai Cancer Center (FUSCC)	TaqMan	mTOR	rs2536 T>C, rs1883965 G>A, rs1034528 G>C, rs17036508 T>C, rs3806317 A>G, rs2295080 T>G	7
Li *et al* (2014) [25]	CGAS	Han	1015/1044	69.1±8.1/68.6 ±8.9	HB	TaqMan	PRKCI	rs546950:T>CRS4955720:C>A	7
Li *et al* (2015) [26]	CGAS	Han	481/480	72.6±9.14/70.0±10.81	Beijing and Tianjin	HRM-PCR	THADA, FOXP4, GPRC6A/RFX6,8q24	THADA-SNP rs1465618, FOXP4-rs1983891, GPRC6A/RFX6-rs339331, 8q24-rs16901966, rs1447295, rs10090154	5
Lin *et al* (2017) [27]	CGAS	Han	I stage: 1343/1008 II stage:1816+1549	I stage:70.6±8.0/62.1±10.0 II stage:69.9±8.2/67.8±6.3	Prostate Cancer Genetics (ChinaPCa) GWAS	MassARRAYiPLEX (Sequenom)	TEX15 MLH3 OR2A5	TEX15-rs142485241 MLH3-rs28756990 OR2A5-rs2961144	5
Liu *et al* (2016) [28]	CGAS	Han	1015/1068	69±8.2/68±9.0	HB	TaqMan	Vav3	rs12410676G>A	7
Liu *et al* (2017) [29]	CGAS	Han, Uygur	413/807	72 ±7.59/72 ±7.65	NA	TaqMan real-time PCR	mTOR Raptor AKTI AKT2 PTEN K-ras	mTOR rs1034528 mTOR rs17036508 mTOR rs12122605 mTOR rs2295080 Raptor rs1468033 Raptor rs2271610 Raptor rs2271612 Raptor rs2292639 Raptor rs3751932 Raptor rs3751934 AKT1 rs2494750 AKT1 rs2494752 AKT2 rs2304186 AKT2 rs7250897 AKT2 rs7254617 PTEN rs701848 K-ras rs7312175	7
Mao *et al* (2014) [30]	CGAS	NA	224/163	NA	HB	microsatellite analysis	AR gene	CAG repeat length	5
Qu *et al* (2016) [31]	CGAS	Han	1817/2026	66.7 ± 7.2/66.9 ± 6.8	HB	SeqMan and Peakscan	MTR MTRR CBS	MTR-rs28372871 MTR-rs1131450 MTR-rs1805087 MTRR-rs326119 MTRR-rs1801394 CBS-rs2850144	7
Liao *et al* (2014) [32]	CGAS	Han	131/229	NA	HB	PCR-RFLP	interleukin-1A (ILIA)	rs3783553	5
Sun *et al* (2021) [33]	CGAS	NA	358/314	70.2/70.9	HB	ABI SNaPshot® multiplex system	RAD17	rs1045051	7
Tao *et al* (2013) [34]	CGAS	Han	665/703	71.5±8.0/71.3±7.9	HB	PCR	CYR61 (IGFBP10) gene	promoter region-rs3753793 (G4T)	6
Wang *et al* (2017) [35]	CGAS	Han	1004/1055	NA	HB	TaqMan-PCR	nucleotide excision repair(NER) genes, XPC, XPD, XPF, and XPG	XPC, rs2228001 T>G and rs1870134 G>C; XPD, rs13181 T>G and rs238406 G>T; XPG, rs1047768 T>C, rs751402 C>T, and rs17655 G>C; and XPF, rs2276464 G>C)	5
Li *et al* (2016) [36]	CGAS	Han	820/945	56.7 ± 12.2/ 56.9 ± 12.7	HB	TaqMan-PCR	NF-κB1	promoter-94 ins/del	6
Wu *et al* (2016) [37]	CGAS	Han	1817/2026	66.7 ± 7.2/66.9 ± 6.8	HB	SNaPshot analysis (ABI)	MTHFR	rs1801133 rs1801131	7
Xu *et al* (2021) [38]	CGAS	Han	165/200	NA	HB	TaqMan	MEG3	rs11627993 and rs7158663	7
Xu *et al* (2012) [11]	GWAS	Han	4484/8934(GWAS) 782/1792(replication 1) 1102/4501(replication 2) 1183/1633(replication 3)	NA	ChinaPCa	MassARRAY iPLEX (Sequenom) or TaqMan (Applied Biosystems)	RAD23B/ KLF4, LILRA3	rs817826 T/C 9q31.2 RAD23B-KLF4 rs103294 T/C 19q13.4 LILRA3 GWAS	6
Zhang *et al* (2016) [39]	GWAS+CGAS	Han	1417/1008(GWAS) 1755/1523(replication)	71.3±8.1/62.1 ± 10.0 and 70.1 ±7.7/67.9±6.3	GWAS+HB	MassARRAY iPLEX (Sequenom)	IGFBP-3	rs969l259 rs6950l79 rs2854744	5
Long *et al* (2012) [40]	CGAS	Han	483/686	67.3 (±6.9)/66.3 (±6.4)	HB	MALDI-TOF mass spectrometry(Sequenom)	C2orf43, FOXP4, GPRC6A/RFX6	C2orf43-rs13385191,rs16988102 FOXP4-rs1983891 GPRC6A/RFX6-rs339331 RFX6-rs9489065 NA-rs12653946 NA-rs9600079	5
Wang *et al* (2012) [41]	GWAS	Han	1524/2169	NA	Prostate Cancer Genetics (ChinaPCa) GWAS	MassARRAY iPLEX(Sequenom)	5 SNPs	C2orf43-rs13385191 NA-rs12653946 FOXP4-rs1983891 GPRC6A/RFX6-rs339331 NA-rs9600079	5
Zhang *et al* (2022) [42]	CGAS	NA	235/252	69.43±9.33/ 68.37±8.47	HB	TaqMan and PCR	GATA2, ZMIZ1, and SUN2	GATA2 (rs73862213, rs2335052, rs10934857) ZMIZ1 (rs704017, rs77911174, rs3740259 SUN2 (rs78397383, rs5750680, rs138705)	7
Zhang *et al* (2014) [43]	CGAS	Han	405/344	NA	DB	HRM-PCR	SNPs	Six variants (rs10505474, rs7837328, rs4242384, rs7813, rs486907 and rs1058205	3
Zhang *et al* (2015) [44]	CGAS	NA	253/214	71.4±7.2/70.2 ±8.0	HB	SNaPshot®	EXO1	rs9350	7
Zhao *et al* (2019) [45]	CGAS	Han	156/188	NA	HB	TaqMan	miR-143/miR-145	Promoter rs4705342	6
Zhao *et al*(2020) [46]	CGAS	Han	218/220	70.54±12.04/ 70.64±11.19	HB	Sanger	GAS5	rs17359906 rs1951625	7
Zhang *et al* (2019) [47]	CGAS	Han	156/188	NA	HB	ligase detection reaction (LDR)	Hsa-miR-23a	rs3745453,rs7372209,rs9660710,rs731384,rs4705342, rs353292	7
Zhou *et al* (2012) [48]	CGAS	Han	193/188	72.78±8.82/ 71.59±8.66	NA	PCR-RFLP	XRCC1	c.910A>G	6
Gu *et al* (2018) [49]	CGAS		917/1036	69.1±7.9/68.7±9.0	HB	TaqMan	ADIPOQ	rs266729 rs182052	5
Li *et al* (2016) [50]	CGAS	Han	168/220	57.76±8.50/ 56.49±9.28	HB	PCR	Estrogen receptor alpha (ESRα)	rs2234693 rs9340799	6
Chen *et al* (2013) [14]	GWAS	Han	1417/1008	NA	Prostate Cancer Genetics (ChinaPCa) GWAS+HapMap Project		GWAS SNP-level analysis-317 SNPs Gene-level analysis-39 Genes	-	4
Cui *et al* (2012) [51]	GWAS	Han	1667/1525	NA	GWAS		-	40 SNPs, 16	4
Rong *et al* (2013) [52]	GWAS	Han	1922/2175	NA	Prostate Cancer Genetics (ChinaPCa) GWAS	Illumina Human OmniExpress Bead Chips, MassARRAY iPLEX(Sequenom)	-	53 SNPs	4
Wu *et al* (2018) [53]	GWAS	Han	1,417 /1,008	NA	Prostate Cancer Genetics (ChinaPCa) GWAS		-	41 CNVs	4

The Genotyping method and Polymorphism data are extracted from previously published sources and remain unchanged. *HB *Hospitals-based data, *DB *Databases-based data

Source*: Indicates the institution or database from which the study participants or data were sourced. HB Hospitalsbased data, DB Databases-based data. Genotyping method*: Specifies the experimental technique used for genetic analysis (e.g., TaqMan: real-time PCRbased allelic discrimination; Sanger: Sanger sequencing). Polymorphism*: Lists specific genetic variants analyzed, including SNP identifiers (rs numbers) or structural variations (e.g., TA repeat sites)

A total of 41 case-control studies reported the association between gene polymorphisms and the incidence of PCa in the Chinese population. All the patients included in the study were Chinese, covering 17 provinces and municipalities across the country. Most of the studies included the Han nationality, only 1 study was about the Yi nationality, and 2 included the Uighur population. The number of participants ranged from 257 to 24,411(7 studies combined GWAS data for analysis) and their mean age varied from 58 to 72.6 years. A total of 116 different polymorphisms (including SNP, deletion, insertion, and repeat length) in 58 genes had been studied in Chinese populations. (Tables 1 and 2) Another 370 single nucleotide polymorphisms (SNPs) and 41 Germline Copy Number Variations (CNVs) were validated in the Genome-wide Association Study (GWAS) of the Chinese population [16, 46, 51, 52] (Table S4). The genetic associations with the PCa epidemiology (findings about individual gene association, gene-gene association, gene-environment association) and the genetic association with the PCa clinical characteristics are further reported in the following.

**Table 2:** Summary of reported basic information from all included studies and estimated quality score for each study

Classification	Candidate Gene	Pathway and function				Main outcome from CGAS in Chinese male						Risk in other population
		Gene Name	Pathway	Function	Reference	Polymorphisms	Effect allele/ reference allele	Analysis	OR, P-value	Outcome	Reference	
**Risk genes**	ZMIZ1	zinc finger MIZ-type containing 1 gene	protein inhibitor of activated STAT (PIAS) family	increase transcriptional activity of androgen receptor	[54]	rs704017	G/A	SHEsis statistical analysis	OR=1.426-2.076, *P*<0.05	Increase PCa risk	Zhang *et al*(2022) [44]	rs77911174 increase PCa risk in Japanese [55]
						rs77911174	G/A	SHEsis statistical analysis	OR=1.351-1.898, *P*<0.05	Increase PCa risk		
						rs3740259	G/A	SHEsis statistical analysis	OR=1.200-1.536, *P*>0.05	No significant association		
	XRCC1	X-ray repair complementing group 1	-	involved in base excision repair	[56]	c.910A>G	G/A	Chi-squared test	OR=1.578-2.870, *P*<0.01	Increase PCa risk	Zhou *et al*(2012) [24]	Increase PCa risk in Caucasian [57]
	TEX15	testis expressed 15, meiosis and synapsis associated	DNA repair gene	repair DNA double-strand break	[58]	rs142485241	G/C	Single-marker association analyses	OR=2.68, *P*=0.0069	Increase PCa risk	Lin *et al*(2017) [28]	-
	SRD5A2	Steroid 5-alpha-reductase 2	Androgen related pathways	Encode testosterone 5-alpha-reductase II, converts testosterone to dihydrotestosteron	[59]	rs523349	C/G	Chi-square test	OR=0.813-0.961, *P*>0.05	No significant association	Luo *et al*(2018) [18]	**Increased risk of PCa in French and Swiss populations ** [60, 61]
						rs9282858	A/G	Chi-square test	OR=1.239-1.250, *P*>0.05	No significant association		**Associations differ among African Americans, Latinos, Caucasians, and Asians ** [62–64]
						TA repeat site	allele (TA)9/allele (TA)0	Chi-square test	OR=2.054-3.697, *P*<0.05	Increase PCa risk		TA repeats influence prostate cancer susceptibility [65]
	Raptor	regulatory associated protein of MTOR complex 1	PI3K/AKT/mTOR signaling pathway	regulates responses to nutrient and insulin levels	[66]	rs1468033	A/G	Chi-square test	OR=1.61, *P*<0.01	Increase PCa risk	Liu *et al*(2017) [31, 67]	-
						rs2271610	C/G	Chi-square test	OR=1.07-1.64, *P*>0.05	No significant association		-
						rs2271612	T/C	Chi-square test	OR=0.76-0.98, *P*>0.05	No significant association		-
						rs2292639	A/C	Chi-square test	OR=0.86-0.94, *P*>0.05	No significant association		-
						rs3751932	T/C	Chi-square test	OR=0.90-0.99, *P*>0.05	No significant association		-
						rs3751934	A/C	Chi-square test	OR=1.02-1.12, *P*>0.05	No significant association		-
	RAD23B/ KLF4	RAD23 homolog B, nucleotide excision repair protein/KLF transcription factor 4	-	-	-	rs817826	C/T	logistic regression analysis	OR=1.41 , *P*=5.45×10-14	Increase PCa risk	Xu *et al*(2012) [11]	-
	RAD17	RAD17 checkpoint clamp loader component	Cell Cycle Checkpoint Gene	mediating DNA damage, ay be involved in homologous recombination	[68]	rs1045051	C/A	Chi-square test	OR=1.296-1.731 , *P*<0.05	Increase PCa risk	Sun *et al*(2021) [34]	-
	OR2A5	olfactory receptor family 2 subfamily A member 5	olfactory receptor (OR) gene family	Associated with neurotransmitter and hormone receptors	[27]	rs2961144	G/A	Single-marker association analyses	OR=1.95 , *P*=0.065	Increase PCa risk	Lin *et al*(2017) [28]	-
	MTR	5-methyltetrahydrofolate-homocysteine methyltransferase	one-carbon metabolism pathway	encoding MTR (a key enzyme in one-carbon metabolism), may increases PCa risk by impairing methylation reactions	[69]	rs28372871	G/T	Univariate and multivariate unconditional logistic regression models	OR=1.13-1.40 , *P*=0.004	Increase PCa risk	Qu *et al*(2016) [32]	MTR 2756 related to a high Gleason score in Spainish [70]
						rs1131450	A/G	Univariate and multivariate unconditional logistic regression models	OR=1.14-1.64, *P*=0.007	Increase PCa risk		
						rs1805087	G/A	Univariate and multivariate unconditional logistic regression models	OR=1.11-1.72, *P*=0.19	No significant association		
	MLH3	mutL homolog 3	DNA repair pathway	Rectifies DNA mismatches during replication and recombination	[71]	rs28756990	A/C	Single-marker association analyses	OR=1.68, *P*=0.06	Increase PCa risk	Lin *et al*(2017) [28]	-
	LILRA3	leukocyte immunoglobulin like receptor A3	LIR family member	regulate immune and inflammatory responses	[72]	rs103294	C/T	logistic regression analysis	OR=1.28, *P*=5.34×10-16	Increase PCa risk	Xu *et al*(2012) [11]	-
	IL-4	interleukin 4	inflammatory response genes	anti-inflammatory role and inhibitor of angiogenesis	[73]	rs2243250	C/T	chi-square test	OR=1.430-2.293, *P*<0.01	Increase PCa risk	Chen *et al*(2016) [19]	-
	COX-2	cyclooxygenase-2	inflammatory response gene	regulate the process of inflammation	[74]	(-1195G>A)	A/G	logistic regression analysis	OR=1.58-2.08, *P*<0.01	Increase PCa risk	Cui *et al*(2015) [21]	**Increased PCa risk in Japanese population;No risk association in Taiwan, United States and Denmark ** [70, 75–78]
						(50C>T)	T/C	logistic regression analysis	OR=1.93 , *P*=0.23	No significant association		-
	miR-23a gene	microRNA 23a	microRNA	Promotes the progression of PCa	[79]	rs7372209	T/C	Unconditional logistic and stratified analyses	OR=0.64-1.23, No *P* value reported	May increase PCa risk	Zhang *et al*(2019) [48]	-
						rs9660710	C/A		OR=1.02-1.48, No *P* value reported	May increase PCa risk		-
						rs731384	T/C		OR=0.72-2.4, No *P* value reported	May increase PCa risk		-
						rs4705342	C/T		OR=1.18-1.36, No *P* value reported	May increase PCa risk		-
						rs353292	T/C		OR=0.26-0.78, No *P* value reported	May increase PCa risk		-
						rs3745453	C/T		OR=1.41-5.23, No *P* value reported	May increase PCa risk		-
	GATA2	GATA2 Gene	GATA transcription factor family	plays an important role in the regulation of androgen receptor (AR) signaling	[80]	rs73862213	G/A	SHEsis statistical analysis	OR=1.734, *P*=0.018	Increase PCa risk	Zhang *et al*(2022) [44]	rs73862213 increase PCa risk in Japanese [55]
						rs2335052	G/A	SHEsis statistical analysis	OR=1.525-1.762, *P*<0.05	Increase PCa risk		-
						rs10934857	A/G	SHEsis statistical analysis	OR=1.12-1.494, *P*>0.05	No significant association		-
	GAS5	growth arrest specific 5	AKT/mTOR signaling pathway	promotes the apoptosis of PCa cells	[81]	rs17359906	A/G	logistic regression analysis	OR=3.44-6.68, *P*<0.001	Increase PCa risk	Zhao *et al*(2020) [16]	-
						rs1951625	A/G	logistic regression analysis	OR=1.38-1.78, *P*<0.05	Increase PCa risk		-
	FOXP4	forkhead box P4	subfamily P of the forkhead box (FOX) transcription factor family	involved in the regulation of tissue- and cell type-specific gene transcription	[82]	rs1983891	T/C	Chi-square test or Fisher’s exact test	OR=1.30-1.418, *P*=4.33×10-8OR(C/T)=0.77,*P*=0.045	Increase PCa risk	Li *et al*(2015) [27] Long *et al*(2012) [41],Wang *et al*(2012) [42]	Increase PCa risk in Japanese [83]
	C2orf43	lipid droplet associated hydrolase(LDAH)	-	encodes lipid droplets protein, associated with prostate cancer	[84]	rs13385191	G/A	logistic regression analysis	OR=1.03-1.33	**Unconfirmed**	Long *et al*(2012) [41],Wang *et al*(2012) [42]	2p24 association with European and American [85]
	EXO1	exonuclease 1	DNA mismatch repair (MMR) pathways	Contributes to modulation of DNA recombination and mediates cell cycle arrest	[86]	rs9350	T/C	logistic regression analysis	OR=1.435-1.955, *P*<0.01	Increase PCa risk	Zhang *et al*(2015) [87]	-
	BIRC5	Baculoviral inhibitor of apoptosis repeat-containing 5	inhibitor of apoptosis protein (IAP) family	apoptotic inhibitor,involves in the apoptosis pathway and cell proliferation	[88]	rs9904341	C/G	logistic regression analysis	OR=1.57, No *P* value reported	May increase PCa risk	Chen *et al*(2013) [20, 51]	-
**Protective gene**	XPC	XPCA complex subunit, DNA damage recognition and repair factor	enetic variants of nucleotide excision repair (NER)	Repairs various types of DNA damage	[89]	rs1870134	C/G	logistic regression analysis	OR=0.75-0.77, *P*<0.01	Decrease PCa risk	Wang *et al*(2017) [37]	**No association found in Asian, Caucasian, or African populations ** [90]
						rs2228001	G/T	logistic regression analysis	OR=0.98-1.13, *P*>0.05	No significant association		
	Vav3	vav guanine nucleotide exchange factor 3	AR and PI3K-AKT signaling pathway	Promotes inflammation and carcinogenesis in prostatic epithelium	[91]	rs12410676	A/G	logistic regression analysis	OR=0.54-0.81, *P*<0.01	Decrease PCa risk	Liu *et al*(2016) [29]	-
	NFKB1	nuclear factor kappa B subunit 1	NF-κB pathway,inflammation and other carcinogenic processes	NF-κB1 and its inhibitor	[92]	Ins/del	Del/Ins	logistic regression analysis	OR=0.57-0.74, *P*<0.05	Decrease PCa risk	Han *et al*(2015) [22] Cui *et al*(2015) [21]	Decrease PCa risk in Denmark
	MTHFR	methylenetetrahydrofolate reductase	folate pathway	causing DNA damage, increased cell death, and inhibiting cell growth.	[93]	rs1801133	T/C	logistic regression analysis	OR=0.68-0.78, *P*=0.0003	Decrease PCa risk	Wu *et al*(2016) [40]	**may be a risk factor of PCa in different ethnic populations ** [94]
						rs1801131	C/A	logistic regression analysis	OR=1.06-1.07, *P*=0.68	No significant association		
	**interleukin-1A (ILIA) **	interleukin-1A	inflammatory response genes	Stimulate tumor cell proliferation	[95]	rs3783553	I/D		OR=0.56, *P*<0.001	Decrease PCa risk	Liao *et al*(2014) [33]	-
	DNMT3B	DNA methyltransferase 3 beta	DNA methylation	Linked to poor prognosis in prostate cancer	[96]	rs2424908	C,G/T	logistic regression analysis	OR=0.57-0.73, *P*<0.01	Decrease PCa risk	He *et al*(2014) [26]	T carriers had reduced risk of a Gleason score in Italian. [96]
	**DNMT1**	DNA methyltransferase 1	DNA methylation	Facilitates prostate tumorigenesis	[97]	rs16999593	C/T	logistic regression analysis	OR=0.41-0.61, *P*<0.05	Decrease PCa risk	He *et al*(2014) [26]	-
						rs2228611	A/G	logistic regression analysis	OR=0.93-0.97, *P*>0.05	No significant association		-
	CYR61 (IGFBP10) gene	cellular communication network factor 1	CCN family	Affects PCa cell morphology, adhesion and proliferation and has pro-tumorigenic effects	[98, 99]	promoter region-rs3753793 (G4T)	G/T	chi-square test	OR=0.73-0.88, No *P* value reported	May decrease PCa risk	Tao *et al*(2013) [35]	-
**Unconfirmed**	mTOR	mechanistic target of rapamycin kinase	PI3K/AKT/mTOR signaling pathway	encodes a protein kinase product of 289 kDa, a critical cell growth effector	[100–102]	rs2536	C/T	logistic regression analysis	OR=0.88-1.45, *P*<0.01	Increase PCa risk	Li *et al*(2013) [50] Liu *et al*(2017) [31, 67]	-
						rs1883965	A/G	logistic regression analysis	OR=1.06-1.337, *P*>0.05	No significant association		-
						rs1034528	C/G	logistic regression analysis	OR=0.93-1.59, *P*<0.01 and *P*>0.05	Inconsistent results		-
						rs17036508	C/T	logistic regression analysis	OR=0.94-3.77, *P*<0.01 and *P*>0.05	Inconsistent results		-
						rs3806317	G/A	logistic regression analysis	OR=0.61-0.93, *P*>0.05	No significant association		-
						rs2295080	G/T	logistic regression analysis	OR=0.54-0.77, *P*<0.01	Decrease PCa risk		-
						rs12122605	T/C	logistic regression analysis	OR=0.98-1.22, *P*>0.05	No significant association		-
	NFKBIA	inflammatory response genes	NF-κB pathway,inflammation and other carcinogenic processes	encodes for IκBα, a protein to inactivate the NF-κB	[103]	rs2233406	T/C	chi-square test	OR=1.188-1.762, *P*>0.05	No significant association	Chen *et al*(2016) [19] Cui *et al*(2015) [21] Han *et al*(2015) [22]	-
						rs3138053	G/A		OR=1.121-1.501, *P*>0.05	No significant association		-
						-826C/T	T/C	chi-square test	OR=0.91-2.83, *P*>0.05 and *P*<0.05	Inconsistent results		-
						3'UTR	G/A		OR=0.91-0.94, *P*>0.05	No significant association		-
						-881AG	G/A		OR=1.34-2.83, *P*<0.01	Increase PCa risk		-
	IL-6	inflammatory response genes	inflammatory response genes	pro-inflammatory cytokine, causes malignant transformation of PCa cells	[104]	rs10499563	C/T	chi-square test	OR=0.504-0.696, *P*<0.01	Decrease PCa risk	Chen *et al*(2016) [19] Huang *et al*(2016) [15]	-
						rs1800796(promotor)	G/C	logistic regression analysis	OR=1.31, No *P* value reported	May increase PCa risk		-
	IGFBP-3	insulin like growth factor binding protein 3	insulin-like growth factor (IGF) axis	Inhibits cell proliferation and induces PCa cell apoptosis	[105]	rs9691259	G/A	logistic regression analysis	OR=0.75-0.85, *P*<0.01	Decrease PCa risk	Zhang *et al*(2016) [47]	**did not influence PCa susceptibility African Americans, Native Hawaiians, Japanese Americans, Latinos, and Whites population ** [106]
						rs6950179	C/T	logistic regression analysis	OR=1.182-1.283, *P*<0.01	Increase PCa risk		
						rs2854744	A/C	logistic regression analysis	OR=1.130-1.418, *P*<0.01	Increase PCa risk		
	GPRC6A/RFX6	G protein-coupled receptor class C group 6 member A/regulatory factor X6	-	GPRC6A:regulates estradiol and testosterone levelsRFX6:encodes a member of the regulatory factor X (RFX) family of transcription factors	[107, 108]	rs339331	T/C	Chi-square test or Fisher’s exact test logistic regression model	OR=0.78-1.34	Inconsistent results	Li *et al*(2015) [27, 49] Long *et al*(2012) [41] Wang *et al*(2012) [42]	Increase PCa risk in Japanese [83]
**NO association**	XPG	ERCC excision repair 5, endonuclease	enetic variants of nucleotide excision repair (NER)	repair of DNA adducts(incision)	[109]	rs17655	C/G	logistic regression analysis	OR=1.17, *P*>0.05	No significant association	Wang *et al*(2017) [37]	-72C/T decrease African Americans PCa risk [109]
						rs751402	T/C	logistic regression analysis	OR=1.00-1.05, *P*>0.05	No significant association		
						rs1047768	C/T	logistic regression analysis	OR=1.07, *P*>0.05	No significant association		
	XPF	ERCC excision repair 4, endonuclease catalytic subunit	enetic variants of nucleotide excision repair (NER)	repair of DNA adducts(incision)	[109]	rs2276464	C/G	logistic regression analysis	OR=0.85-0.90, *P*>0.05	No significant association	Wang *et al*(2017) [37]	-
	XPD	ERCC excision repair 2, TFIIH core complex helicase subunit	enetic variants of nucleotide excision repair (NER)	repair of DNA adducts(DNA uncoili)	[109]	rs13181	G/T	logistic regression analysis	OR=0.64-1.19, *P*>0.05	No significant association	Wang *et al*(2017) [37]	**Association with Gleason score and PCa stage in Caucasians and African Americans ** [110]
						rs238406	T/G	logistic regression analysis	OR=1.13-1.24, *P*>0.05	No significant association		
	THADA	THADA armadillo repeat containing	-	unclear	[111]	rs1465618	A/G	Chi-square test or Fisher’s exact test	OR=1.13-1.24, *P*>0.05	No significant association	Li *et al*(2015) [27, 49]	**Increased PCa risk in UK and Australians ** [112]
	SUN2	SAD1/UNC84 domain protein-2	-	key component of the linker of nucleoskeleton and cytoskeleton (LINC) complex.Loss of promotes the progression of prostate cancer by regulating fatty acid oxidation	[113]	rs78397383	C/A	SHEsis statistical analysis	OR=0.992-1.062, *P*>0.05	No significant association	Zhang *et al*(2022) [44]	**rs138708 increase PCa risk in Japanese ** [55]
			-			rs5750680	T/C	SHEsis statistical analysis	OR=1.105-1.145, *P*>0.05	No significant association		
			-			rs138705	T/C	SHEsis statistical analysis	OR=1.02, *P*>0.05	No significant association		
	PTEN	phosphatase and tensin homolog	PI3K/AKT/mTOR signaling pathway	suppress tumor cell growth, invasion and metastasis	[114]	rs701848	C/T	chi-square test	OR=0.87-0.93, *P*>0.05	No significant association	Liu *et al*(2017) [31, 67]	-
	PRKCI	protein kinase C iota	-	encodes atypical protein kinase C, aunique human oncoprotein	[115]	rs546950	C/T	logistic regression analysis	OR=1.06-1.10, *P*>0.05	No significant association	Li *et al*(2015) [49]	**Decrease PCa risk in European ** [116]
						rs4955720	A/C	logistic regression analysis	OR=0.91-1.14, *P*>0.05	No significant association		
	PPARG	peroxisome proliferator activated receptor gamma	inflammatory response gene	encodes for peroxisome proliferator-activated receptor-γ (PPAR-γ)	[117]	Pro12Ala	Ala/pro	logistic regression analysis	OR=1.05-1.09, *P*>0.05	No significant association	Cui *et al*(2015) [21]	**Associated with increased PCa risk only in individuals with high BMI (American) ** [118]
	PCA3	prostate cancer antigen 3	lncRNA(regulate nearby genes and distant transcriptional factors)	Expressed in prostate tissue and related with malignant transformation of prostatic epithelial cells and with higher Gleason score	[119–121]	rs544190	A/G	logistic regression analysis	OR=0.98-1.1289, *P*>0.05	No significant association	Cao *et al*(2018) [17]	**PCA3 -845 G>A increase PCa risk in white men(Portuguese) ** [122]
	NF-κB1	Nuclear factor-κB	NF-κB signalling pathway	Promotes tumor growth by triggering inflammation and blocking cell death.	[123]	promoter-94 ins/del	Del/Ins	Unconditionallogistic regression analysis	OR=0.95-1.27, No *P* value reported	No significant association	Li *et al*(2016) [38, 43]	-
	MTRR	5-methyltetrahydrofolate-homocysteine methyltransferase reductase	one-carbon metabolism pathway	catalyzes the regeneration of methylcobalamin, keep MTR active	[69]	rs326119	C/A	Univariate and multivariate unconditional logistic regression models	OR=0.86-0.91, *P*=0.31	No significant association	Qu *et al*(2016) [32]	-
						rs1801394	G/A	Univariate and multivariate unconditional logistic regression models	OR=0.93-0.99, No *P* value reported	No significant association		-
	miR-143/miR-145	microRNA 143/microRNA 145	microRNA	Increase sensitivity of PCa to radiotherapy	[124]	rs4705342	C/T	Univariate and/or multivariate logistic regression analysis	OR=2.4, No *P* value reported	No significant association	Zhao *et al*(2019) [23]	-
	MEG3	maternally expressed 3	Long Non-Coding RNA	Inhibit prostate cancer cell proliferation and promote cell apoptosis	[67]	rs11627993a1	A/C	logistic regression analysis	OR=1.03-1.30, No *P* value reported	No significant association	Xu *et al*(2020) [39]	-
						rs7158663a2	G/A	logistic regression analysis	OR=0.86-1.97, No *P* value reported	No significant association		-
	K-ras	KRAS proto-oncogene, GTPase	PI3K/AKT/mTOR signaling pathway	Facilitates the onset and progression of PCa	[125]	rs7312175	A/G	Chi-square test	OR=1.07-1.18, *P*=0.238	No significant association	Liu *et al*(2017) [31, 67]	-
	KLK3	kallikrein 3	-	Encode PSA protein	[126]	rs1058205	homozygous/heterozygous	Chi-squared test	OR=0.7-1.57, *P*>0.05	No significant association	Chen *et al*(2017) [127, 128]	**Associated with lower serum PSA in Swedish and African American men ** [129, 130]
	IL-8	inflammatory response genes	inflammatory response genes	pro-inflammatory cytokine,implicates carcinogenic processes	[131]	rs4073	A/T	chi-square test	OR=0.762-0.912, *P*>0.05	No significant association	Chen *et al*(2016) [19]	**Decreased PCa risk in UK study ** [132]
	COX-1	cyclooxygenase-2	inflammatory response gene	regulate the process of inflammation	[133]	(50C>T)	T/C	logistic regression analysis	OR=0.98-1.31, *P*=0.1	No significant association	Cui *et al*(2015) [21]	-
	CBS	cystathionine beta-synthase	the transsulfurization pathway	A gene encoding a rate-limiting enzyme that catalyzes homocysteine in the transsulfurization pathway, related to DNA methylation	[134]	rs2850144	G/C	Univariate and multivariate unconditional logistic regression models	OR=0.96-0.98, No *P* value reported	No significant association	Qu *et al*(2016) [32]	-
	AR gene	androgen receptor	Androgen related pathways	Closely related to the development and treatment of prostate cancer	[135]	CAG repeat length		t test and Chi-squared test		17 CAG repeats may be associated with TMPRSS2:ERG fusion positive PCa	Mao *et al*(2014) [36]	-
	AKT2	AKT serine/threonine kinase 2	PI3K/AKT/mTOR signaling pathway	promotes cell migration by downregulating GSK3b	[136]	rs2304186	T/G	Chi-square test	OR=0.91-1.00, *P*>0.05	No significant association	Liu *et al*(2017) [31, 67]	-
						rs7250897	C/T	Chi-square test	OR=1.11-1.29, *P*>0.05	No significant association		-
						rs7254617	A/G	Chi-square test	OR=0.93-2.00, *P*>0.05	No significant association		-
	AKT1	AKT serine/threonine kinase 1	PI3K/AKT/mTOR signaling pathway	activation inhibits cell migration	[137]	rs2494750	C/G	Chi-square test	OR=0.89-0.97, *P*>0.05	No significant association	Liu *et al*(2017) [31, 67]	-
						rs2494752	G/A	Chi-square test	OR=0.87-1.01, *P*>0.05	No significant association		-
	ADIPOQ	adiponectin, C1Q and collagen domain containing	adipokine	Encodes adiponectin, restrains PCa cell proliferation and invasion	[138]	rs266729	G/C	logistic regression analysis	OR=0.96-1.02, *P*>0.05	No significant association	Gu *et al*(2018) [45]	**Associated with the risk of PCa in the Caucasian population ** [139]
						rs182052	A/G	logistic regression analysis	OR=0.86-0.90, *P*>0.05	No significant association		
	GEMIN4	gem nuclear organelle associated protein 4	-	Involved in the maturation process of miRNA, target RNA recognition and inhibition	[140]	rs7813	T/C	Chi-square test	OR=0.9-1.1, *P*>0.05	No significant association	Zhang *et al*(2014) [25]	**Increase PCa risk in Chinese ** [141]
	RNASEL	ribonuclease L	-	regulating cell proliferation and apoptosis	[142]	rs486907	G/A	Chi-square test	OR=1.27-1.68, *P*>0.05	No significant association	Zhang *et al*(2014) [25]	**AA gene reduces PCa risk among Ashkenazi Jews ** [143]
Others	Estrogen receptor alpha (ESRα)	Estrogen receptor alpha	estrogen-related pathway	May involve recurrence and progression in prostate cancer patients	[144]	rs2234693	T/C	Chi-square test	X2=6.328	ORR, PFS better in TTgenotype	Li *et al*(2016) [38, 43]	**Associations differ among US, Indian, Slovak, Japanese populations ** [50]
						rs9340799	A/G	Chi-square test	X2=9.334	ORR, PFS better in A allele carriers	Li *et al*(2016) [38, 43]	
Unmapped	5p15(region)	-	-	-	-	rs12653946	T/C	logistic regression analysis	OR=1.30-1.41, *P*=4.43×10-8	Increase PCa risk	Long *et al*(2012) [41] Wang *et al*(2012) [42]	Increase PCa risk in Japanese [83]
	13q22(region)	-	-	-	-	rs9600079	T/G	logistic regression analysis	OR=1.07-1.18, *P*<0.01 and P unkwon	Inconsistent results	Wang *et al*(2012) [42]	Increase PCa risk in Japanese [83]
	8q24(region) 8q24(region) 8q24(region)	chromosomal region	-	Related to aggressive PCa	[145]	rs16901966	G/A	Chi-square test or Fisher’s exact test	OR=0.66, No *P* value reported	May decrease PCa risk	Li *et al*(2015) [27, 49]	-
						rs1447295	A/C	Chi-square test or Fisher’s exact test	OR=1.46, No *P* value reported	May increase PCa risk		Increased PCa risk in African and European populations [146]
						rs10090154	T/C	Chi-square test or Fisher’s exact test	OR=0.58, No *P* value reported	May decrease PCa risk		-
		-	-	-	-	rs7837328	A/G	Chi-square test	OR=1.38-1.93, *P*<0.01	Increase PCa risk	Zhang *et al*(2014) [25]	-
		-	-	-	-	rs10505474	A/G	Chi-square test	OR=0.79-1.56, *P*<0.01	Increase PCa risk	Zhang *et al*(2014) [25]	-
	NA	-	-	-	-	rs4742384	C/A	Chi-square test	OR=0.57-1.31, *P*>0.05	No significant association	Zhang *et al*(2014) [25]	-
	NA	-	-	-	-	rs1058205	C/T	Chi-square test	OR=0.87-1.09 *P*>0.05	No significant association	Zhang *et al*(2014) [25]	-

Bolded text indicates that the candidate gene/polymorphism is associated with inconsistent PCa risk in Chinese and other populations

### Genetic association with the incidence of PCa

#### Individual gene association with the incidence of PCa (Table 2 and Supplementary Table 1)

Table 2 summarizes the effects of 116 polymorphisms across 58 candidate genes on the overall risk of PCa in the Chinese population and, more importantly, presents the metabolic pathways and functions of these genes. Additionally, this table shows the differences in the findings on these polymorphisms from other studies, highlighting the potential impact of candidate genes on PCa risk in different populations.

According to the studies included in this review, 36 variants in 24 genes (XPG, XPF, XPD, THADA, SUN2, PTEN, PRKCI, PPARG, PCA3, NF-κB1, MTRR, miR-143/miR-145, MEG3, K-ras, KLK3, IL-8, COX-1, CBS, AR gene, AKT2, AKT1, ADIPOQ, GEMIN4, RNASEL) and 2 unmapped SNPs failed to show association with PCa while other polymorphisms had a negative or positive effect (Table 2). Odds ratios (ORs) were estimated for risk variants about the risk of PCa.

Regarding the increased risks of PCa, among the genes identified above, 29 of 40 polymorphisms in the 20 genes (ZMIZ1, XRCC1, TEX15, SRD5A2, Raptor, RAD23B/ KLF4, RAD17, OR2A5, MTR, MLH3, LILRA3, IL-4, COX-2, Hsa-miR-23a, GATA2, GAS5, FOXP4, C2orf43, EXO1, BIRC5) and 5 unmapped SNPs (rs12653946C > T, rs9600079 G > T, rs1447295 C > A, rs7837328 G > A, rs10505474 G > A) were shown to increase PCa risk in Chinese population, conferring odds ratio (OR) ranged from 1.28–6.68 [16, 18–22, 24, 26–29, 31–35, 37, 40–42, 44, 47, 48]. Genotypes of GAS5 (rs17359906 G > A, OR = 6.68) [16], Hsa-miR-23a (rs3745453 T > C, OR = 4.16–5.23; rs4705342 T > C, OR = 2.06–2.4) [48], SRD5A2 (TA repeat site G > C, OR = 2.05–3.7) [18], XRCC1(c.910 A > G, OR = 2.29–2.87), IL-4 (rs2243250 T > C, OR = 2.29) [19], ZMIZ1 (rs704017 A > G, OR = 2.076) [44], TEX15 (rs142485241 C > G, OR = 2.68) [28] increased the risk of PCa in China by more than 2 times.

Regarding the reduced risks of PCa, 8 of 11 polymorphisms in the 8 genes (XPC, Vavs, NFKB1, MTHFR, interleukin-1A (ILIA), DNMT3B, DNMT1, CYR61 (IGFBP10)) 2 unmapped SNPs (rs16901966 A > G, rs10090154 C > T) were shown to be associated with reduced risk in men with PCa, conferring odds ratio (OR) ranged from 0.41–0.88. Genotypes of DNMT1 (rs2228611 G > A, OR = 0.41) [26] and ILIA (rs3783553 D/I or I/I, OR = 0.48) [33] were the most significant reduction in PCa risk.

Polymorphisms in 8 genes (FOXP4, C2orf43, NFKB1, mTOR, NFKBIA, IL-6, IGFBP-3, GPRC6A/RFX6) were investigated repeatedly in at least two studies. The effect of FOXP4 (rs1983891 C > T, OR = 1.3–1.41) and NFKB1 (Ins/Del, OR = 0.69–0.74) on PCa risk in 3 studies was consistent [27, 41, 42].Interestingly, these studies were not without conflicting findings. For instance, 5 genes (mTOR, NFKBIA, IL-6, GPRC6A/RFX6, IGFBP-3) showed different effects on PCa risk in different studies. Both Li et al. (2013) and Liu et al. (2017) reported the association of mTOR rs1034528 G > C, rs17036508 T > C, and rs2295080 T > G with PCa in a Chinese population. Both articles believed that rs2295080 T > G (adjusted OR = 0.54–0.77, *P* < 0.05) was related to the reduction of PCa incidence in China. However, mTOR rs1034528 G > C (adjusted OR = 1.31, *P* = 0.005) was considered to be associated with an increased risk of PCa in the study by Li et al. (2013) [50], while the study of Liu et al. (2017) did not report any association (adjusted OR = 0.93–1.59, P > 0.1) [31].

Another inconsistent finding about the genetic association of PCa was noted when Li et al. (2013) reported that rs17036508 T > C was not associated with PCa susceptibility (adjusted OR = 0.94–1.23, *P* > 0.05) [50], while Liu et al. (2017) believed that it significantly increased the risk of PCa (adjusted OR = 3.73, *P* = 0.001) [31]. Likewise, Cui et al. (2015) found that NFKBIA −826C/T (adjusted OR = 0.91–1.09) was not associated with the risk of PCa [19], while Han et al. (2015) believed that it was significantly associated with an increased risk of PCa (adjusted OR = 1.84–2.83, *P* < 0.05) [22].

The three studies by Li et al. (2015), Long et al. (2012), and Wang et al. (2012) about the relationship between GPRC6A/RFX6 rs339331 and the incidence of PCa also reached different conclusions [27, 41, 42], which reported reduced risks (OR = 0.78, *P* = NA), increased risks (OR = 1.34, *P* = NA) and unrelated association (OR = 0.99, *P* = 0.84) respectively. The association of C2orf43 (rs13385191A > G) with the risk of PCa in Long et al. (2012) [41]and Wang et al. (2012) [42]are also controversial, which indicated increased risk (OR = 1.33 *P* = NA) and irrelevant association (OR = 1.03, *P* = 0.48) respectively.

When comparing the GWAS, it was common for the studies to select SNPs from the reported GWAS in the European ancestry, African-American, and Japanese populations to verify the genetic factors of PCa among the Chinese population (Supplementary Table 5). One of these studies showed the associations between PCa and Germline Copy Number Variations (CNVs). Chen et al. (2013) [51]proposed 4 genes (EGFR, ERBB2, PTK2, and RAF1) with five SNPs (rs11238349, rs17172438, rs984654, rs11773818, and rs17172432) as the key factors from 39 genes and 317 SNPs influencing PC. On the other hand, Cui et al. (2012) and Rong et al. (2013) screened 16 and 24 SNPs from 40 and 53 SNPs [46, 53], respectively, which were suggested to be associated with the risk of PCa in the Chinese population. Wu et al. (2018) found that [46], among 41 CNVs, 27 CNVs were risk variations, and the other 14 were found to be protective of PC. Xu et al. (2012) performed the first GWAS in Han Chinese and identified two new risk-associated loci for PCa on chromosomes 9q31.2 (rs817826, *P* = 5.45 × 10^−14^) and 19q13.4 (rs103294, *P* = 5.34 × 10^−16^) in 4,484 PCa patients [11].

There were also reports about the genetic association with the PCa treatment outcome. Li et al. (2016) found that the ESRα gene (rs2234693 and rs2234693) was also considered to be associated with PCa risk [43]. TT genotype in rs2234693 or GG genotype in rs9340799 might be associated with a higher overall response rate (ORR) in chemotherapy, while T allele and A allele carriers had significantly longer progression-free survival (PFS).

#### Gene-gene association with the incidence of PCa (Supplementary Table 2)

Some studies reported gene-gene association with PCa incidence. Eight studies reported the effect of gene-gene interaction in 13 SNPs on the risk of PCa (Supplementary Table 2). Four publications by Liu et al. (2017), Long et al. (2012), Wang et al. (2012), and Zhang et al. (2014) respectively found that when there were more than 2 risk loci or risk alleles, the risk of PCa in the carrier was significantly increased (OR = 1.39–3.50, Ptrend < 0.001) [31, 41–43]. Additionally, in the combined analysis, some SNPs had a slightly reduced PCa risk. The combined effect of rs546950 and rs4955720 in PRKCI reduced the risk of PCa in carriers (OR = 0.63, *P* = 0.045) [25]. Moreover, individuals carrying two SNPs of Vav3 rs12410676(A) and rs8676(A) had a protective effect in < 69 years old, (OR = 0.66, *P* = 0.022) and stage I + II, (OR = 0.68, *P* = 0.014) [29].

#### Gene-environment association with the incidence of PCa(Supplementary Table 3)

Various non-gene factors were shown to have an association with the risks of PCa such as age, race, family history, smoking, obesity, and alcohol consumption. Among the included studies, 16 reported that 24 SNPs in 17 genes were associated with 7 environmental factors (age at diagnosis, Smoking status, Alcohol status, BMI, ethnicity, Family history, Complications) to pose positive or negative effects on the risk of PCa (Supplementary Table 2). On the other hand, 7 other SNPs (Raptor rs1468033 G > A, PRKCI rs4955720 C > A, MTHFR rs1801131 C > A, AKT2 rs7250897 T > C, ADIPOQ rs266729 C > G, rs182052 G > A, miR-143/miR-145 rs4705342 T > C) were shown to be not associated with the overall risk of PCa but had positive or negative effects in smokers/non-smokers, High/Low BMI, older/younger subjects, ever drinking, and Uygur ethnicity.

In the older populations, 12 polymorphisms in 8 genes (NFKBIA, MTR, mTOR, Hsa-miR-23a, EXO1, Raptor, AKT2, GAS5) were associated with increased PCa risk. The odds ratio ranged from 1.33–5.62, and GAS5 rs17359906 G > A was the highest [16, 22, 31, 32, 47, 48]. In contrast, NFKB1 9-4ATTG,-del/del (OR = 0.57) and XPCA rs1870134 G > C (OR = 0.78) [37] were protective factors. In the younger populations, 7 variants and 4 genes (GAS5, mTOR, MTR, NFKBIA) were associated with increased PCa risk. The odds ratio ranged from 1.4–4.75, and mTOR rs17036508 T > C was the highest [16, 22, 31, 32]. In contrast, ADIPOQ rs182052 G > A(OR = 0.73) [49], PRKCI rs4955720 C > A (OR = 0.45) [25] and mTOR rs2295080 T > G (OR = 0.67) [31]were considered to reduce the risk.

Among smokers, 13 polymorphisms in 13 gene (COX-2, NFKBIA, miR-143/miR-145, Raptor, Vavs, GAS5, mTOR, Hsa-miR-23a, NFKB1) were associated with increased PCa risk. The odds ratio ranged from 1.32–6.39, and mTOR rs17036508T > C was the highest [16, 21, 22, 29, 31, 45, 48, 50].In contrast, mTOR rs2295080 T > G (OR = 0.42–0.72) [31] and NFKB1 9–4ATTG del/del (OR = 0.42) [22] were protective factors. In non-smokers, 5 variants in 4 genes (NFKBIA, IL6, Raptor, GAS5) were considered to increase the risk of PCa (OR = 1.38–4.78, highest: GAS5 rs17359906G > A) [22], while PRKCI rs4955720 C > A (OR = 0.43 and XPCA rs1870134 G > C (OR = 0.75) were considered to reduce the risk [25, 31, 37].

In populations with high BMI, 9 polymorphisms in 6 genes (Hsa-miR-23a, mTOR, MTR, Raptor, AKT2, GAS5) were associated with increased PCa risk. The odds ratio ranged from 1.47 −8.11, and mTOR rs17036508T > C was the highest [16, 31, 32, 48, 50]. In contrast, mTOR rs2295080 T > G (OR = 0.43–0.8) [31, 37] was protective factor. In low BMI populations, 3 polymorphisms in 3 genes (XPC, Vavs, PRKCI) were considered to decrease the risk of PCa (OR = 0.51–0.78, lowest: PRKCI rs4955720 C > A) [25, 29, 31, 37, 50], while some SNPs in mTOR and GAS5 were considered to increase the risk (OR = 1.29–3.89, highest: GAS5 rs17359906G > A) [16, 31, 50].

In terms of alcohol intake, in ever or never-drinking populations, 5 SNPs in 3 genes (GAS5, miR-143/miR-145, Hsa-miR-23a) were considered to increase the risk (OR = 1.75–8.19, highest: miR-143/miR-145 rs4705342 T > C) [16, 45, 48]. Regarding ethnic factors, 2 studies reported that in the Uighur population, Raptor rs1468033 G > A (OR = 1.66) and mTOR rs17036508 T > C (OR = 5.09) were associated with increased PCa risk [31, 50]. For people with a family history of cancer, Zhang et al. (2019) reported that Hsa-miR-23a rs3745453 T > C increased the risk of PCa in people without a family history of cancer (OR = 1.89) [48].

Regarding populations with co-morbidities, Qu et al. (2016) reported that some SNPs in MTR increased the risk of PCa in people with hypertension, diabetes mellitus, and cardiovascular disease (OR = 1.35–4.17, highest: rs1805087 G > A) [32]. In contrast, rs1801133 T > C in MTHFR was associated with decreased PCA risk(0.66–0.8) in people with hypertension, diabetes mellitus, and cardiovascular disease, while rs1801131 C > A increased the risk (OR = 6.49) [40].

#### Genetic association with the clinical characteristics of PCa (Supplementary Table 4)

A total of 24 SNPs in 16 genes (EXO1, IL6, mTOR, RAD17, CYR61 (IGFBP10), Vavs, MTHFR, MTR, GAS5, PCA3, PRKCI, DNMT1, DNMT3B, XPC, BIRC5, KLK3) were reported to be associated with the overall disease stage (e.g. localized or advanced disease), Gleason score(high/low), PSA levels, PCa risk, clinicopathological characteristics (e.g. seminal vesicle invasion, positive surgical margin, lymph node involvement, extracapsular extension, aggressive disease) in Chinese with PCa (Supplementary Table 4).

Regarding the disease stage of PCa, subgroups of localized diseased, 3 SNPs in 3 genes (EXO1, IL6, RAD17) were reported to increase risk of PCa (OR = 1.66–3.08, highest: EXO1 C > T) [23, 34, 47], while mTOR rs2295080 T > G decreased the risk. In patients with advanced diseases, 3 SNPs in EXO1 and mTOR had a positive association with PCa risk (OR = 1.5–1.91, highest: mTOR rs2536 T > C), while CYR61 rs3753793 T > G had an opposite effect (OR = 0.7) [35].

In the subgroups of high Gleason score, 7 SNPs in 3 genes (MTR, GAS5, mTOR) were associated with an increased risk of PCa (OR = 1.3–4.54, highest: GAS5 rs1951625 G > A) [16, 32, 50],while mTOR rs2295080 T > G, Vavs rs12410676 G > A and CYR61 rs1801133T > C had an opposite effect (OR = 0.53–0.79) [29, 35, 40, 50]. In subgroups of low Gleason score, 4 SNPs in 4 genes (PRKCI, DNMT1, DNMT3B, XPC) were associated with a decreased risk of PCa (OR = 0.38–0.72, lowest: PRKCI rs4955720 C > A) [25, 26, 37], while the other 4 SNPs in PC3 and mTOR had a positive association (OR = 1.25–1.58) [17, 50]. IL6 rs1800796 C > G was associated with an increased risk in PCa patients with Gleason score = 7 [15].

In terms of PSA levels, 3 SNPs in GAS5 and BIRC5 had a positive association (OR = 1.08–4.079, highest: GAS5 rs17359906 G > A) in PCA patients with high(> 20 ng/ml) or low(PSA ≤ 20 ng/ml) PSA levels [16, 20]. Regarding clinicopathological characteristics, SNPs in MTR were reported to associate with a decreased risk of PCa in subgroups of seminal vesicle invasion, positive surgical margin (NO), Lymph node involvement (YES), extracapsular extension, and aggressive disease [32]. Moreover, rs1801133 T > C in MTHFR was considered to have a negative association in the subgroup of seminal vesicle invasion, positive surgical margin (NO), Lymph node involvement(YES), extracapsular extension, aggressive disease (OR = 0.43–0.87), while rs1801131 C > A showed positive effect(OR = 3.38) [40]. Reportedly, Vavs rs12410676 G > A was also associated with aggressive disease in PCa patients [29]. PCa risk KLK3 rs1058205 C > T was reported to have a positive association in PCA patients with moderate risk and high risk(OR = 2.0–3.08) [147].

## Discussion

According to the 41 genetic studies included in this systematic review, the correlation between genetic factors and PCa susceptibility in Chinese populations mainly involved 7 GWAS and, to a greater extent, 34 CGAS. Firstly, this study analyzed 37 polymorphisms in 28 candidate genes and 7 unmapped SNPs identified in the Chinese population, revealing both positive and negative impacts on PCa risk. This enhances the current understanding of key risk alleles in Asian populations. Secondly, this study found that among the 116 polymorphisms across 58 candidate genes included in all CGAS studies, the results for 18 candidate genes/polymorphisms differed from those in other populations. Additionally, 29 SNPs validated by GWAS showed inconsistent associations with PCa risk between the Chinese population and other ethnic groups. These findings collectively highlight the unique genetic background of PCa in the Chinese population. This is particularly significant because previous GWAS have predominantly focused on men of European descent, comprising 78% of all GWAS participants, whereas Asian men represented only 11% despite Asians making up 59.7% of the global population [127].

Thirdly, by integrating GWAS and CGAS information specific to the Chinese population, this study offers a comprehensive exploration of the candidate genes and pathways responsible for observed differences, thereby facilitating further investigation into the physiological significance of these genes in Chinese PCa. Previously, although over 200 genetic loci associated with PCa risk variants have been identified globally through GWAS, less than half of the familial risk has been elucidated. Therefore, the biological significance of many PCa susceptibility loci remains unclear based solely on GWAS results. The integrative analysis of data from GWAS and CGAS in this study explains single nucleotide polymorphisms and lineage mutations which may serve as an important reference for determining the risk and further explaining the epidemiological and disease characteristics of PCa in China.

### Comparing genetic influences on prostate cancer across different populations

Among the 116 polymorphisms from 58 candidate genes analyzed in our study, 28 candidate genes have been validated in males of other ethnicities. Among these, 18 candidate genes/polymorphisms exhibit differences in PCa risk between Chinese males and males of other ethnicities (Table 2). Specific polymorphisms in genes such as SRD5D2, XPC, XPD, THADA, SUN2, PRKCI, PPARG, PCA3, KLK3, IL-8, ADIPOQ, GEMIN4, and RNASEL have been linked to PCa risk in European, Japanese, American, and African populations, showing either positive or negative effects [57, 61–65, 94, 106, 112, 116, 118, 122, 129, 130, 132, 139, 141, 143, 148]. However, these associations are not observed in the Chinese population. Notably, polymorphisms in COX-2 (−1195A/G) and IGFBP-3 (rs6950179C/T) increase PCa risk in the Chinese population, whereas no such association is observed in American, European, Taiwanese, or Japanese populations [75–78, 85, 146]. Conversely, the MTHFR (rs1801133T/C) polymorphism is associated with a reduced PCa risk in the Chinese population, while no significant association is observed in Caucasians. Interestingly, some studies suggest that the MTHFR (C677T) polymorphism may be linked to an increased risk of PCa in East Asians. However, whether these differences translate into physiological effects requires further investigation.

Additionally, three GWAS reports on PCa risk-associated SNPs identified 386 SNPs correlated with PCa risk in Chinese males. Among these, 45 SNPs were identified as potentially associated with PCa risk in Chinese males, with 40 SNPs previously validated in European and Japanese populations. Nevertheless, 29 SNPs previously validated in European and Japanese populations as potentially related to PCa risk did not demonstrate a correlation with PCa risk in Chinese males. These findings are consistent with previous research, further highlighting potential differences in genetic background and susceptibility to PCa in the Chinese population compared to other ethnic groups [100].

### Candidate genes with significant effect on prostate cancer in China

Among the 116 polymorphisms in 58 candidate genes, 28 genes with 37 polymorphisms and 7 unmapped SNPs exerted either positive or negative effects on the risk of PCa in the Chinese population. The significance of the study findings is at least 2-fold. Of the 29 polymorphisms in 20 candidate genes that increased the risk of PCa, only 8 polymorphisms in 6 genes increased the risk by more than 2-fold among Chinese males, while the rest had odds ratios (OR) less than 2.

Among the 6 genes associated with a more than 2-fold increased risk of PCa in the Chinese population, ZM1Z1 and SRD5A2 are related to Androgen-related pathways, TEX15 is a DNA repair gene, IL-4 is involved in inflammatory responses, miR-23a is a microRNA, and GAS5 is related to the AKT/mTOR signaling pathway. Conversely, genes with the most pronounced protective effects (OR less than 0.5) against PCa in Chinese males include DNMT1 and ILIA, which are associated with DNA methylation and inflammation, respectively.

It is also worth noting that SNPs showing inconsistent effects on PCa risk in Chinese populations across different studies are concentrated in the mTOR, NFKBIA, IL-6, and IGFBP-3 genes, potentially associated with the PI3K/AKT/mTOR signaling pathway, inflammation, and other carcinogenic processes, as well as apoptosis promotion and anti-angiogenesis [83, 101–104, 149].

### Additive risks of genetic factors

In this study, additive risks of PCa were identified when considering the interaction between different genes, and the interaction between gene and environment. Regarding the gene-gene interaction, a trend where the cumulative effect of SNPs contributes to an elevated risk of PCa among Chinese males was identified in this study. This trend predominantly involves genes associated with inflammatory response (COX1-COX2, NFKBIA) and those related to the PI3K/AKT/mTOR signaling pathway (Vav3, mTOR, AKT2). These findings parallel those of Takata et al. [150],where each variant exhibited an odds ratio (OR) ranging from 1.11 to 1.31, indicating a moderate association with PCa risk. Notably, males carrying 5–6 risk alleles showed a 2.19-fold increase in the risk of developing PCa compared to those carrying 0–2 risk alleles, underscoring the collective impact of independent risk loci in PCa occurrence.

Regarding the gene-environment interaction, within the studies included in our analysis, we observed a synergistic effect of genetic and environmental factors (such as smoking status, BMI, age, drinking status, ethnicity, family history, and disease) on the risk of PCa in Chinese males. Specifically, PRKCI rs4955720 C > A, AKT2 rs7250897 T > C, and ADIPOQ rs182052 G > A were found to have no independent effect on the risk of PCa in Chinese males. However, when PRKCI rs4955720 C > A interacted with non-smokers or individuals with low BMI, the risk of PCa in Chinese males was significantly reduced (OR = 0.43–0.51). Conversely, when AKT2 rs7250897 T > C interacted with high BMI or older subjects, the risk of PCa in Chinese males significantly increased (OR = 1.58–1.83). These findings suggest that the incidence of PCa in China may be the result of a combination of genetic background and lifestyle factors. Such findings aligned with previous studies which observed variations in PCa risk among different generations of Asian immigrants, indicating the importance of genetic background and lifestyle factors in PCa occurrence [5].

### Genetic factors and clinical attributes

This study found that candidate genes and polymorphisms may be associated with PCa clinical characteristics in addition to affecting the overall risk of PCa. In previous studies, it has been observed that a larger proportion of PCa patients in China exhibit higher levels of PSA and Gleason scores, as well as poorer prognosis compared to Caucasian populations [54, 128].This suggests that there may be racial differences in the biological characteristics of PCa between Chinese and Caucasian populations. In our study, we identified 16 genes with 23 SNPs that play positive or negative roles in different disease biology characteristics such as disease stage, Gleason score, PSA levels, PCa risk, and clinicopathological characteristics. This indicates that the observed biological differences between Chinese and Caucasian populations may be attributed to genetic factors. The candidate genes mTOR and GAS5 related to the PI3K/AKT/mTOR signaling pathway were the most frequently occurring genes and may be associated with advanced disease and high Gleason scores in Chinese PCa patients.

### Recommendations for future research

In summary, we conducted a thorough and systematic assessment of the existing evidence regarding genetic factors in Chinese male PCa. We synthesized information on candidate genes and susceptibility variants linked to PCa in Chinese males based on available data. Nevertheless, larger-scale GWAS involving a larger Chinese population and mechanistic studies of candidate genes are needed to deepen our understanding of PCa susceptibility and genetic mechanisms in China.

Based on our systematic review, we underscore the current status of genetic factors influencing PCa risk and provide insights into recommendations for future research. Our review identifies potential candidate genes for further mechanistic studies, aimed at elucidating the biological mechanisms by which genes impact PCa risk. This exploration could uncover potential targets for screening, diagnosis, and even treatment. Additionally, validation through further research with large sample sizes from diverse ethnic populations is crucial. Furthermore, there are ongoing developments in PCa genetic risk scoring, aimed at assessing disease susceptibility, identifying high-risk individuals, and stratifying advanced PCa cases. To provide comprehensive information on the role of genetic background in PCa incidence across different racial groups, more genomic research is needed.

### Strengths and limitations of this review

One of the primary strengths of this review is the breadth of our literature search. We conducted systematic searches of both English and Chinese databases, ensuring comprehensive coverage of relevant literature. Additionally, our analysis encompasses both candidate gene association studies (CGAS) and genome-wide association studies (GWAS), offering a comprehensive overview of genetic factors in Chinese male PCa. Furthermore, all studies included in our review employed a case-control design, comparing PCa cases with non-PCa male controls. This approach enables us to draw meaningful conclusions from the current data, summarizing candidate genes and susceptibility variants associated with PCa in the Chinese male population.

In discussing the limitations of our study, several key points should be addressed. Firstly, due to constraints regarding the number of articles and scope, we had to exclude articles published before 2012 and studies with fewer than 100 PCa patients. This exclusion may have led to the oversight of potentially significant findings. Secondly, our study did not incorporate meta-analyses and focused solely on case–control studies, thereby potentially missing out on comprehensive comparative analyses of potential genetic loci. Thirdly, our research solely examined genetic factors associated with the overall PCa risk, without further analysis of genetic factors' impact on prognosis and response to drug therapy. Moreover, among the 41 articles included in our study, only 7 were GWAS studies, analyzing 1417–4484 PCa patients. The remaining 34 CGAS studies had considerably smaller sample sizes, ranging from 122 to 1004 patients. This disparity in sample size compared to genetic studies in other populations might have influenced the robustness of our results. Additionally, the contradictory results for a few genes across different studies may be attributed to the influence of sample size.

## Conclusion

In conclusion, our systematic review comprehensively compiled and analyzed genetic susceptibility research on PCa in Chinese populations, identifying 28 genes and 44 polymorphisms significantly associated with PCa, as well as 45 potential loci from GWAS screening. These findings provide crucial insights into the relationship between genetic susceptibility and PCa risk in Chinese men, highlighting the importance of gene-gene and gene-environment interactions in shaping PCa risk. Further research is essential to unravel these complex mechanisms and their role in the development of PCa in China.

## Supplementary Information

Supplementary Material 1

## Data Availability

Data availability statements All data generated or analysed during this study are included in this published article [and its supplementary information files].
