# Supplementary material for: Genetic associations of prostate cancer in China: a systematic review
Source: BMC Cancer. 2025 Apr 3;25:604. doi: 10.1186/s12885-025-13830-9 (PMC11966891; doi:10.1186/s12885-025-13830-9)
Supplement: Supplementary file 1 — Supplementary Material 1 [file 12885_2025_13830_MOESM1_ESM.docx]

**Supplementary Figure 1. Study designs of included publications.**

**Supplementary Table 1. Summary of Gene and Polymorphism Classifications in Prostate Cancer Risk Assessment.**

| Classification | Risk genes | Protective genes | Unconfirmed | No significant association | Unmapped | Others | Total |
| --- | --- | --- | --- | --- | --- | --- | --- |
| Genes | 20 | 8 | 5 | 24 | 0 | 1 | 58 |
| Polymorphisms | 40 | 11 | 18 | 36 | 9 | 2 | 116 |
| Polymorphisms(Effective) | 29 | 8 | 12 | 0 | 7 | 2 | 58 |
| Polymorphisms (Invalid) | 11 | 3 | 6 | 36 | 2 | 0 | 58 |

**Supplementary Table 2. Gene-gene association with the incidence of PCa.**

| **Author(Years)** | **Genetic polymorphisms** | | **OR,95% CI** | **Effect** |
| --- | --- | --- | --- | --- |
|  | **Gene 1** | **Gene 1** |  |  |
| Cui et al(2015) | COX-2 (-1195AA) | COX-1 (50CT) | OR = 1.83, 95% CI = 1.06–3.17, P = 0.03) | increased |
| Han et al(2015) | NFKBIA-826CT | NFKBIA-881AG | OR = 0.36-0.49 | increased |
| Li et al(2014) | PRKCI-rs546950 | PRKCI-rs4955720 | adjusted OR = 0.63, 95% CI = 0.40–0.99, P = 0.045 | decreased |
| Li et al(2015) | FOXP4-rs1983891, GPRC6A/RFX6-rs339331, 8q24-rs16901966, rs1447295, rs10090154 | | 2 risk alleles: OR=1.69 (1.06-2.56) ,P=0.017 ≥3risk alleles: OR=1.99(1.30-3.03) ,P=0.001 | increased |
| Liu et al(2016) | Vav3-rs12410676 (G>A) | Vav3-rs8676(G>A) | <69 years old, (OR=0.66,P=0.022) stage I+II, (OR=0.68,P=0.014) | decreased |
| Liu et al(2017) | mTOR rs17036508 mTOR rs2295080 Raptor rs1468033 | AKT2 rs7250897 | Ptrend<0.001 2 genotypes: Adjusted OR=2.04(1.31-3.18), P=0.002 3 genotypes: Adjusted OR=3.50(2.27-5.38), P<0.001 4 genotypes: Adjusted OR=2.07(1.30-3.28), P=0.002 >1genotypes: Adjusted OR=2.56(1.71-3.84), P<0.001 | increased |
| Mao et al(2014) | AR gene-CAG repeat length | | 17 CAG repeats, P=0.037 |  |
| Long et al(2012) | C2orf43-rs13385191  NA-rs12653946 FOXP4-rs1983891 GPRC6A/RFX6-rs339331 | | rs13385191*rs16988102, OR=1.71 (1.47–1.96) 4 SNPs, OR=2.06(1.79-2.35) | increased |
| Wang et al(2012) | C2orf43-rs13385191, 5p15-rs12653946, FOXP4-rs1983891, RFX6-rs339331, 13q22-rs9600079 | | Ptrend = 2.58 × 10-13 3 OR=1.43 (1.21–1.69), P=3.95x10-5 4 OR=1.62 (1.35–1.94), P=1.52x10-7 5–6 OR=2.26 (1.78–2.87), P=1.98x10-11 | increased |
| Zhang et al(2014) | rs7837328 (A) | rs10505474 (A) | Ptrend=2.58×10-5 2 risk allele : OR=1.39(1.07-1.79), P=0.01 >=3 isk allele : OR=1.58(1.18-2.11), P=0.001 | increased |

**Supplementary Table 3. Gene-environment association with the incidence of PCa.**

| **Risk factor** | **Stratification** | **Gene** | **Findings** | **Effect** | **Author(Years)** |
| --- | --- | --- | --- | --- | --- |
| Smoking status | Smokers | COX-2 | -1195G>A, OR = 1.76, 95% CI = 1.28–2.41, P < 0.01 | increase | Cui et al(2015) |
|  |  | NFKBIA | -826C>T, OR=2.78; 95% CI: 1.46, 5.31; P<0.01  -881A>G, OR=2.78; 95% CI: 1.46, 5.31; P<0.01 | increase | Han et al(2015) |
|  |  | **miR-143/miR-145** | **rs4705342 T>C, OR=5.29( 1.11 -25.19),P=0.02(recessive model)** | **increase** | **Zhao et al(2019)** |
|  |  | **Raptor** | **rs1468033 G>A, Adjusted OR=1.58(1.13-2.19), P=0.007** | **increase** | **Liu et al(2017)** |
|  |  | Vavs | rs12410676 G>A, OR=0.69,P=0.002(recessive model) | increase | Liu et al(2016) |
|  |  | GAS5 | rs17359906G>A,OR= 3.43 (1.76-6.66) ,P= <0.001(dominant model) | increase | Zhao et al(2020) |
|  |  | mTOR | rs2536 T>C, OR=1.42 (1.06–1.92), P=0.021 rs1034528 G>C, OR=1.32 (1.04–1.67),P=0.023 rs2295080 T>G, OR=0.72 (0.57–0.91) ,P=0.006 rs17036508T>C, Adjusted OR=6.39(2.46-16.60), P<0.001(recessive model) rs2295080 T>G, Adjusted OR=0.42(0.22-0.80), P=0.008(dominant model) | opposite | Li et al(2013), Liu et al(2017) |
|  |  | Hsa-miR-23a | rs3745453T>C,OR = 2.22, 95% CI = 1.12–4.41(recessive model) | increase | Zhang et al(2019) |
|  |  | NFKB1 | 9–4ATTG, del/del, OR=0.42; 95% CI: 0.22, 0.80; P<0.01 | decrease | Han et al(2015) |
|  | Non-smokers | **PRKCI** | **rs4955720 C>A,OR=0.43 (0.20–0.92), P= 0.030** | **decrease** | **Li et al(2014)** |
|  |  | NFKBIA | -826 C>T, OR=1.38; 95% CI: 1.07, 1.78; P=0.01(heterozygotes) -826 C>T, OR= 3.24; 95% CI: 1.75, 6.02; P<0.01(mutants) -881 A>G, OR=1.38; 95% CI: 1.07, 1.78; P=0.01(heterozygotes) -881 A>G, OR= 3.24; 95% CI: 1.75, 6.02; P<0.01(mutants) | increase | Han et al(2015) |
|  |  | IL6 | rs1800796,C>G OR = 1.85, 95%CI = 1.07-3.22,P = 0.029(dominant model) | increase | Huang et al(2016) |
|  |  | **Raptor** | **rs1468033 G>A, Adjusted OR=1.61(1.12-2.30), P=0.009** | **increase** | **Liu et al(2017)** |
|  |  | GAS5 | rs17359906G>A,OR= 4.78 (2.70-8.42) ,P= <0.001(dominant model) | increase | Zhao et al(2020) |
|  |  | XPC | rs1870134 G>C, OR=0.75 (0.60-0.94), P=0.012(dominant model) | decrease | Wang et al(2017) |
| BMI | High BMI | Hsa-miR-23a | rs3745453T>C, OR = 2.04, 95% CI = 1.17–3.58(recessive model) | increase | Zhang et al(2019) |
|  |  | mTOR | rs17036508T>C, Adjusted OR=8.11(2.62-25.11), P<0.001(recessive model) rs2295080 T>G,Adjusted OR=0.43(0.22-0.86), P=0.016(dominant model) rs2295080 T>G, OR=0.68 (0.49–0.94) ,P=0.021 | opposite | Li et al(2013), Liu et al(2017) |
|  |  | MTR | rs28372871G>T,OR= 1.47 (1.07–2.01),P=0.016(dominant model) | increase | Qu et al(2016) |
|  |  | **Raptor** | **rs1468033 G>A, Adjusted OR=2.02(1.42-2.87), P<0.001(dominant model) rs1468033 G>A,Adjusted OR=5.13(1.94-13.6), P=0.001(recessive model)** | **increase** | **Liu et al(2017)** |
|  |  | **AKT2** | **rs7250897 T>C, Adjusted OR=1.58(1.12-2.24)(dominant model)** | **increase** | **Liu et al(2017)** |
|  |  | GAS5 | rs17359906G>A,OR= 4.07 (2.05- 8.11) ,P= <0.001(dominant model) | increase | Zhao et al(2020) |
|  | Low BMI | XPC | rs1870134 G>C, OR=0.70 (0.56-0.86), P=0.0007(dominant model) | decrease | Wang et al(2017) |
|  |  | Vavs | rs12410676 G>A, OR=0.78,P=0.025(recessive model) | decrease | Liu et al(2016) |
|  |  | mTOR | rs2536 T>C, OR=1.74 (1.30–2.32) P=0.0002 rs1034528 G>C, OR=1.47 (1.17–1.84) ,P=0.001 rs2295080 T>G, OR=0.79 (0.64–0.98) ,P=0.0351 rs17036508T>C,OR=1.29 (1.01–1.66) ,P=0.042(dominant model) rs17036508T>C, Adjusted OR=3.20(1.20-8.53), P=0.020(recessive model) | opposite | Li et al(2013), Liu et al(2017) |
|  |  | **PRKCI** | **rs4955720 C>A,OR=0.51 (0.29–0.90), P= 0.019** | **decrease** | **Li et al(2014)** |
|  |  | GAS5 | rs17359906G>A,OR= 3.89 (2.21-6.84) ,P= <0.001(dominant model) | increase | Zhao et al(2020) |
| Age | Older subjects | NFKB1 | 9–4ATTG,-del/del,OR=0.57; 95% CI: 0.32, 0.99; P=0.05 | decrease | Han et al(2015) |
|  |  | NFKBIA | -826C>T(OR=1.33; 95% CI: 1.02, 1.72; P=0.03 -881A>G(OR=2.98; 95% CI: 1.67, 5.33; P<0.01 | increase | Han et al(2015) |
|  |  | MTR | rs28372871G>T,OR= 1.42 (1.04–1.96),P=0.028(dominant model) | increase | Qu et al(2016) |
|  |  | mTOR | rs2295080 T>G, OR=0.74 (0.57–0.96) ,P=0.024(dominant model) rs2295080 T>G, Adjusted OR=1.46(1.06-2.00), P=0.021(recessive model) rs17036508T>C, Adjusted OR=4.88(1.65-14.38), P=0.004(recessive model) | opposite | Li et al(2013), Liu et al(2017) |
|  |  | XPC | rs1870134 G>C, OR=0.78 (0.63-0.97) , P=0.023(dominant model) | decrease | Wang et al(2017) |
|  |  | Hsa-miR-23a | rs3745453T>C, OR = 1.86, 95% CI = 1.01–3.41(recessive model) | increase | Zhang et al(2019) |
|  |  | EXO1 | rs9350 C>T, OR=1.758 (1.008–3.064), P=0.047(heterozygotes) rs9350 C>T, OR=1.776 (1.051–3.002), P=0.032(dominant model) rs9350 T allele, OR=1.464 (0.998–12.147) , P=0.041 | increase | Zhang et al(2015) |
|  |  | Raptor | rs1468033 G>A, Adjusted OR=1.81(1.31-2.48), P<0.001 | increase | Liu et al(2017) |
|  |  | **AKT2** | **rs7250897T>C, Adjusted OR=1.83(1.33-2.52), P<0.001(dominant model)** | **increase** | **Liu et al(2017)** |
|  |  | GAS5 | rs17359906G>A,OR= OR= 5.62 (3.21-9.81) ,P= <0.001(dominant model) | increase | Zhao et al(2020) |
|  | Younger subjects | GAS5 | rs17359906G>A,OR= 2.40 (1.22-4.70) ,P= 0.016(dominant model) | increase | Zhao et al(2020) |
|  |  | **ADIPOQ** | **rs182052G>A,Adusted OR=0.73 (0.54-0.99),P=0.04(recessive model)** | **decrease** | **Gu et al(2018)** |
|  |  | XPC | rs1870134 G>C, OR=0.73 (0.54-0.98) , P= 0.037(dominant model) | decrease | Wang et al(2017) |
|  |  | mTOR | rs2536 T>C, OR=1.42 (1.03–1.95), P= 0.031 rs1034528 G>C,OR=1.40 (1.09–1.81),P= 0.010 rs2295080 T>G, Adjusted OR=0.67(0.46-0.97), P=0.035(recessive model) rs17036508T>C, Adjusted OR=4.75(1.78-12.71), P=0.002(recessive model) rs17036508T>C,Adjusted OR=1.81(1.19-2.77), P=0.006 | opposite | Li et al(2013), Liu et al(2017) |
|  |  | **PRKCI** | **rs4955720C>A, OR=0.45 (0.23–0.87), P= 0.018(dominant model)** | **decrease** | **Li et al(2014)** |
|  |  | MTR | rs1131450 A>G, OR=1.77 (1.08–2.88) , P= 0.022(dominant model) | increase | Qu et al(2016) |
|  |  | NFKBIA | -826C>T,OR=2.69; 95% CI: 1.34, 5.42; P=0.01  -881A>G,OR=2.69; 95% CI: 1.34, 5.42; P=0.01 | increase | Han et al(2015) |
| Driking status | Ever Drinking | GAS5 | rs17359906G>A,OR= 4.05 (1.89-8.68) ,P= <0.001(dominant model) | increase | Zhao et al(2020) |
|  |  | **miR-143/miR-145** | **rs4705342 T>C, OR=8.19(0.98 -68.71),P=0.03(recessive model)** | **increase** | **Zhao et al(2019)** |
|  | Never Drinking | GAS5 | rs17359906G>A,OR= 4.04 (2.40-6.80) ,P= <0.001(dominant model) rs1951625G>A, OR= 1.75(1.10-2.79) ,P= 0.025(dominant model) | increase | Zhao et al(2020) |
|  |  | Hsa-miR-23a | rs3745453T>C, OR = 2.04, 95% CI = 1.13–3.68(recessive model) | increase | Zhang et al(2019) |
| Ethnicity | Uygur | **Raptor** | **rs1468033 G>A, Adjusted OR=1.66(1.26-2.20), P<0.001** | **increase** | **Liu et al(2017)** |
|  | Uygur | mTOR | rs17036508T>C, Adjusted OR=5.09(2.20-11.78), P<0.001(recessive model) | increase | Li et al(2013) |
| Family history | no family history of cancer | Hsa-miR-23a | rs3745453T>C, OR = 1.83, 95% CI = 1.11–3.01(recessive model) | increase | Zhang et al(2019) |
| Disease | Hypertension | MTR | rs28372871G>T,OR=1.45 (1.11–1.90), P=0.006(dominant model) rs1805087 G>A,OR= 4.17 (1.10–15.83), P= 0.026(dominant model) | increase | Qu et al(2016) |
|  |  | MTHFR | rs1801133 T>C, OR=0.66 (0.49–0.90), P=0.007(dominant model) | decrease | Wu et al(2016) |
|  | Diabetes mellitus | MTR | rs28372871G>T,OR= 1.35 (1.13–1.61), P=9 x10-4(dominant model) rs1131450 A>G, OR=1.55 (1.08–2.21), P=0.017(dominant model) | increase | Qu et al(2016) |
|  |  | MTHFR | rs1801133 T>C, OR= 0.66 (0.49–0.90), P=0.008(dominant model) | decrease | Wu et al(2016) |
|  | Cardiovascular disease | MTR | rs28372871G>T,OR=2.32 (1.2–4.25),P=0.006(dominant model) rs1131450 A>G, OR=1.80 (1.26–2.58), P=0.001(dominant model) rs1805087 G>A,OR= OR= NA (0.00-NA), P= 0.005(dominant model) | increase | Qu et al(2016) |
|  |  | **MTHFR** | rs1801133 T>C, OR= 0.80 (0.66–0.98), P=0.027(dominant model) **rs1801131 C>A, OR=6.49 (1.08–39.01), P=0.028(dominant model)** | opposite | Wu et al(2016) |

**Supplementary Table 4. Genetic association with the clinical characteristics of PCa.**

| **Risk factor** | **Stratification** | **Gene** | **Findings** | **Effect** | **Author(Years)** |
| --- | --- | --- | --- | --- | --- |
| Disease stage | Localized disease | EXO1 | rs9350 C>T, OR=3.077 (1.440-6.573), P=0.004 rs9350 C>T, OR=1.798 (1.070-3.022), P= 0.027(dominant model) rs9350 T allele, OR=1.678 (1.175-2.396) , P=0.004 | increased | Zhang et al(2015) |
|  |  | IL6 | rs1800796C>G, OR = 1.66, 95%CI = 1.04-2.66, P = 0.034(dominant model) | increased | Huang et al(2016) |
|  |  | mTOR | rs2295080 T>G,OR=0.69 (0.55–0.88) ,P=0.002(dominant model) | decreased | Li et al(2013) |
|  |  | RAD17 | rs1045051A>C,OR=2.423 (1.286-4.566), P=0.006 | increased | Sun et al(2021) |
|  | Advanced disease | EXO1 | rs9350 C>T, OR=1.749 (1.124-2.723) , P=0.013(heterozygotes) | increased | Zhang et al(2015) |
|  |  | mTOR | rs2536 T>C,OR=1.91 (1.47–2.49),P <0.0001(dominant model) rs1034528 G>C,OR=1.53 (1.22–1.91) P=0.0002(dominant model) rs17036508 T>C,OR=1.50 (1.18–1.91),P=0.001(dominant model) | increased | Li et al(2013) |
|  |  | CYR61 (IGFBP10) | rs3753793T>G,P=0.012, Adjusted OR=0.70 (0.53–0.94)(dominant model) | decreased | Tao et al(2013) |
| Gleason score | High Gleason score | Vavs | rs12410676 G>A,OR=0.79,P=0.028(dominant model) | decreased | Liu et al(2016) |
|  |  | CYR61 (IGFBP10) | rs3753793T>G,P=0.002, Adjusted OR=0.63 (0.46–0.86)(dominant model) | decreased | Tao et al(2013) |
|  |  | MTHFR | rs1801133T>C, OR= 0.53 (0.39–0.71), P=3 × 10-5(dominant model) | decreased | Wu et al(2016) |
|  |  | MTR | rs28372871G>T, OR=1.67 (1.31–2.14),P=<0.0001(dominant model) rs1131450 A>G, OR=2.83 (1.78–4.48), P<0.0001(dominant model) rs1805087 G>A,OR= 2.79 (1.13–6.87), P =0.028(dominant model) | increased | Qu et al(2016) |
|  |  | GAS5 | rs1951625 G>A,OR= 1.87(1.07-3.25),P=0.038(dominant model) rs1951625 G>A,OR= 4.54(1.92-10.71) ,P<0.001 | increased | Zhao et al(2020) |
|  |  | mTOR | rs2536 T>C,OR=1.44 (1.11–1.87) P=0.007(dominant model) rs1034528 G>C,OR=1.30 (1.05–1.60) ,P=0.016(dominant model) rs2295080 T>G,OR=0.75 (0.61–0.93) ,P=0.008(dominant model) | opposite | Li et al(2013) |
|  | Low Gleason score | PCA3 | rs544190 G>A, OR=1.254, P=0.046(dominant model) | increased | Cao et al(2018) |
|  |  | mTOR | rs2536 T>C,OR=1.58 (1.15–2.18) P=0.005(dominant model) rs1034528 G>C,OR=1.51 (1.16–1.96) ,P=0.002(dominant model) rs17036508 T>C,OR=1.44 (1.08–1.92) ,P=0.013(dominant model) | increased | Li et al(2013) |
|  |  | PRKCI | rs4955720C>A, OR=0.38 (0.16–0.90), P= 0.028(dominant model) | decreased | Li et al(2014) |
|  |  | DNMT1 | rs16999593T>C(OR=0.47,95% CI 0.28~0.85,P=0.008)(dominant model) | decreased | He et al(2014) |
|  |  | DNMT3B | rs2424908C>G, OR=0.46,95% CI 0.26-0.85, P=0.009)(dominant model) | decreased | He et al(2014) |
|  |  | XPC | rs1870134 G>C,OR=0.72 (0.56-0.93), P=0.012(dominant model) | decreased | Wang et al(2017) |
|  | Gleason score=7 | IL6 | rs1800796C>G, OR = 1.82, 95%CI = 1.11-3.00, P = 0.019(dominant model) | increased | Huang et al(2016) |
| PSA levels | PSA<=20ng/ml | GAS5 | rs17359906 G>A,OR=4.07(1.63-10.16),P= 0.004(heterozygotes) rs1951625 G>A, OR=1.08(1.01-1.08),P=0.033(heterozygotes) | increased | Zhao et al(2020) |
|  | > 20 ng/ml | BIRC5 | rs9904341G>C,adjusted OR = 1.62, 95% CI = 1.12-2.33,P = 0.01(dominant model) | increased | Chen et al(2013) |
| PCa risk | moderate risk | KLK3 | rs1058205 C>T, OR=2.000; 95% CI: 1.205–3.322 | increased | Chen et al(2017) |
|  | high risk | KLK3 | rs1058205 C>T, OR=3.676; 95% CI: 1.080–12.50 | increased | Chen et al(2017) |
| Clinicopathological characteristics | Seminal vesicle invasion | MTR | rs28372871G>T , OR=1.29 (1.08–1.54) ,P= 0.006(dominant model) rs1131450 A>G, OR=3.08 (1.83–5.18),P<0.0001(dominant model) | increased | Qu et al(2016) |
|  |  | MTHFR | rs1801133T>C, OR=0.43 (0.29–0.62), P=2 × 10-5(dominant model) | decreased | Wu et al(2016) |
|  | Positive surgical margin(NO) | MTHFR | rs1801133T>C, OR=0.82 (0.67–1.00) , P=0.045(dominant model) | decreased | Wu et al(2016) |
|  |  | MTHFR | rs1801133T>C, OR= 0.69 (0.47–1.00) , P=0.046(dominant model) | decreased | Wu et al(2016) |
|  |  | MTR | rs1131450 A>G, OR=1.51 (1.05–2.16),P=0.026(dominant model) | increased | Qu et al(2016) |
|  | Lymph node involvement(YES) | MTR | rs28372871G>T , OR=1.96 (1.27–3.02),P= 0.003(dominant model) rs1131450 A>G, OR=3.02 (1.39–6.57),P=0.008(dominant model) | increased | Qu et al(2016) |
|  |  | MTHFR | rs1801133T>C, OR= 0.13 (0.05–0.32), P= 1×10-6(dominant model) rs1801131C>A,OR= 3.38 (1.41–8.13), P= 0.011(dominant model) | opposite | Wu et al(2016) |
|  |  | MTHFR | rs1801133T>C, OR=0.87 (0.72–1.04), P=0.13(dominant model) | decreased | Wu et al(2016) |
|  |  | MTR | rs28372871G>T , OR=1.23 (1.04–1.47), P=0.017(dominant model) rs1131450 A>G, OR= 1.45 (1.02–2.05),P=0.039(dominant model) | increased | Qu et al(2016) |
|  | Extracapsular extension | MTR | rs28372871G>T ,OR=2.08 (1.62–2.67) ,P<0.0001(dominant model) rs1131450 A>G, OR=2.41 (1.51–3.87),P=3×10-4(dominant model) | increased | Qu et al(2016) |
|  |  | MTHFR | rs1801133T>C, OR= 0.51 (0.38–0.70), P=8 × 10-5(dominant model) | decreased | Wu et al(2016) |
|  | Aggressive disease | Vavs | rs12410676 G>A,OR=0.80,P=0.022(dominant model) | decreased | Liu et al(2016) |

**Supplementary Table 5.** **Summary of SNP and CNV Variants in Chinese Populations from GWAS Data**

| **Variant Type** | **Polymorphisms (SNP)** | **CHR** | **BP / Start-End (CNV)** | **Minor Allele** | **Locus/Genes** | **OR** | **P-value** | **CNV Type** | **Direction** | **Associated with PCa risk** | **Origin of GWAS** | **Reference** |
| --- | --- | --- | --- | --- | --- | --- | --- | --- | --- | --- | --- | --- |
| SNP | rs1465618 | 2 | 43407453 | G | THADA | 0.86 | 0.02 | - | - | YES | Europeans | Cui et al(2012) |
| SNP | rs721048 | 2 | 62985235 | A | EHBP1 | 1.36 | 0.042 | - | - | YES | Europeans | Cui et al(2012) |
| SNP | rs12621278 | 2 | 173019799 | G | ITGA6 | 0.86 | 0.019 | - | - | YES | Europeans | Cui et al(2012) |
| SNP | rs7679673 | 4 | 106280983 | C | TET2 | 1.18 | 0.021 | - | - | YES | Europeans | Cui et al(2012) |
| SNP | rs12653946 | 5 | 1948829 | T | - | 1.2 | 1.87E-03 | - | - | YES | Japanese | Cui et al(2012) |
| SNP | rs339331 | 6 | 117316745 | C | - | 0.83 | 1.92E-03 | - | - | YES | Japanese | Cui et al(2012) |
| SNP | rs1512268 | 8 | 23582408 | A | NKX3.1 | 1.23 | 9.39E-04 | - | - | YES | Europeans | Cui et al(2012) |
| SNP | rs10086908 | 8 | 128081119 | C | - | 0.78 | 9.24E-04 | - | - | YES | Europeans | Cui et al(2012) |
| SNP | rs16901979 | 8 | 128194098 | A | - | 1.44 | 5.14E-09 | - | - | YES | Europeans | Cui et al(2012) |
| SNP | rs1447295 | 8 | 128554220 | A | - | 1.38 | 7.04E-06 | - | - | YES | Europeans | Cui et al(2012) |
| SNP | rs10993994 | 10 | 51219502 | C | MSMB | 0.89 | 0.038 | - | - | YES | Europeans | Cui et al(2012) |
| SNP | rs10896449 | 11 | 68751243 | G | - | 1.37 | 0.032 | - | - | YES | Europeans | Cui et al(2012) |
| SNP | rs902774 | 12 | 51560171 | A | - | 0.5 | 0.035 | - | - | YES | Europeans | Cui et al(2012) |
| SNP | rs9600079 | 13 | 72626140 | T | - | 1.18 | 3.97E-03 | - | - | YES | Japanese | Cui et al(2012) |
| SNP | rs11649743 | 17 | 33149092 | T | HNF1B | 0.85 | 8.51E-03 | - | - | YES | Europeans | Cui et al(2012) |
| SNP | rs5759167 | 22 | 41830516 | T | TTLLI.BIK | 0.83 | 5.99E-03 | - | - | YES | Europeans | Cui et al(2012) |
| SNP | rs16901979 | 8q24(Region2) | 128194098 | A | - | 1.48 | 2.33E-14 | - | - | YES | European | Rong et al(2013) |
| SNP | rs1447295 | 8q24(Region1) | 128554220 | A | LOC727677 | 1.48 | 1.54E-08 | - | - | YES | European | Rong et al(2013) |
| SNP | rs6983267 | 8q24(Region3) | 128482487 | G | - | 1.34 | 4.55E-10 | - | - | YES | European | Rong et al(2013) |
| SNP | rs1512268 | 8p21 | 23582408 | T | NKX3-1 | 1.34 | 8.26E-09 | - | - | YES | European | Rong et al(2013) |
| SNP | rs103294 | 19q13.4 | 59489660 | C | LILRA3 | 1.34 | 3.15E-08 | - | - | YES | Chinese | Rong et al(2013) |
| SNP | rs817826 | 9q31.2 | 109196121 | C | - | 1.49 | 0.000000826 | - | - | YES | Chinese | Rong et al(2013) |
| SNP | rs12653946 | 5p15 | 1948829 | T | - | 1.26 | 0.00000154 | - | - | YES | Japanese | Rong et al(2013) |
| SNP | rs9600079 | 13q22 | 72626140 | T | - | 124 | 0.0000035 | - | - | YES | Japanese | Rong et al(2013) |
| SNP | rs339331 | 6q22 | 117316745 | T | RFX6 | 123 | 0.0000291 | - | - | YES | Japanese | Rong et al(2013) |
| SNP | rs4430796 | 17q12 | 33172153 | A | HNF1B | 1.2 | 0.000515 | - | - | YES | European | Rong et al(2013) |
| SNP | rs620861 | 8q24(Region4) | 128335673 | G | - | 128 | 0.00163 | - | - | YES | European | Rong et al(2013) |
| SNP | rs1465618 | 2p21 | 43407453 | T | THADA | 1.17 | 0.00354 | - | - | YES | European | Rong et al(2013) |
| SNP | rs6763931 | 3q23 | 142585523 | A | ZBTB38 | 115 | 0.00438 | - | - | YES | European | Rong et al(2013) |
| SNP | rs2252004 | 10q26 | 122834699 | C | - | 1.17 | 0.00442 | - | - | YES | Japanese | Rong et al(2013) |
| SNP | rs721048 | 2p15 | 62985235 | A | EHBP1 | 1.39 | 0.0114 | - | - | YES | European | Rong et al(2013) |
| SNP | rs12621278 | 2q31 | 173019799 | A | ITGA6 | 114 | 1.47E-02 | - | - | YES | European | Rong et al(2013) |
| SNP | rs11649743 | 17q12 | 33149092 | G | HNF1B | 111 | 0.0315 | - | - | YES | European | Rong et al(2013) |
| SNP | rs5759167 | 22q13 | 41830156 | G | BIK | 1.12 | 0.0329 | - | - | YES | European | Rong et al(2013) |
| SNP | rs10875943 | 12q13 | 47962277 | C | - | 115 | 0.0356 | - | - | YES | European | Rong et al(2013) |
| SNP | rs887391 | 19q13 | 46677464 | T | - | 1.1 | 0.0366 | - | - | YES | European | Rong et al(2013) |
| SNP | rs13385191 | 2p24 | 20751746 | G | C2orf43 | 1.1 | 0.0417 | - | - | YES | Japanese | Rong et al(2013) |
| SNP | rs10486567 | 7p15 | 27943088 | G | JAZF1 | 115 | 0.0429 | - | - | YES | European | Rong et al(2013) |
| SNP | rs6465657 | 7q21 | 97654263 | C | LMTK2 | 1.14 | 0.0477 | - | - | YES | European | Rong et al(2013) |
| SNP | rs9364554 | 6q25 | 160753654 | C | SLC22A3 | 11 | 0.0483 | - | - | YES | European | Rong et al(2013) |
| SNP | rs10993994 | 10q11 | 51219502 | T | MSMB | 1.12 | 0.0589 | - | - | NO | European | Rong et al(2013) |
| SNP | rs7127900 | 11p15 | 2190150 | G | - | 119 | 0.0617 | - | - | NO | European | Rong et al(2013) |
| SNP | rs10086908 | 8q24(Region5) | 128081119 | T | - | 1.12 | 0.0761 | - | - | NO | European | Rong et al(2013) |
| SNP | rs7679673 | 4q24 | 106280983 | C | TET2 | 111 | 0.0839 | - | - | NO | European | Rong et al(2013) |
| SNP | rs10187424 | 2p11 | 85647808 | T | - | 1.08 | 0.129 | - | - | NO | European | Rong et al(2013) |
| SNP | rs130067 | 6p21 | 31226490 | G | CCHCR1 | 11 | 0.14 | - | - | NO | European | Rong et al(2013) |
| SNP | rs10896449 | 11q13 | 68751243 | G | - | 1.16 | 1..43E-01 | - | - | NO | European | Rong et al(2013) |
| SNP | rs1983891 | 6p21 | 41644405 | T | FOXP4 | 1.07 | 2.13E-01 | - | - | NO | Japanese | Rong et al(2013) |
| SNP | rs10936632 | 3q26 | 171612796 | A | CLDN11 | 1.09 | 2.59E-01 | - | - | NO | European | Rong et al(2013) |
| SNP | rs2121875 | 5p12 | 44401302 | A | FGF1- | 0.94 | 2.72E-01 | - | - | NO | European | Rong et al(2013) |
| SNP | rs8102476 | 19q13 | 43427453 | C | PPP1R14A | 1.05 | 2.84E-01 | - | - | NO | European | Rong et al(2013) |
| SNP | rs10934853 | 3q21 | 129521063 | A | EEFSEC | 1.05 | 3.04E-01 | - | - | NO | European | Rong et al(2013) |
| SNP | rs2928679 | 8p21 | 23494920 | A | SLC25A37 | 1.07 | 0.345 | - | - | NO | European | Rong et al(2013) |
| SNP | rs1938781 | 11q12 | 58671686 | G | FAM111A | 1.05 | 0.351 | - | - | NO | Japanese | Rong et al(2013) |
| SNP | rs16902094 | 8q24.21 | 128320346 | A | - | 1.07 | 0.455 | - | - | NO | European | Rong et al(2013) |
| SNP | rs2660753 | 3p12 | 87193364 | T | - | 1.04 | 0.467 | - | - | NO | European | Rong et al(2013) |
| SNP | rs1859962 | 17q24 | 66620348 | G | - | 1.03 | 0.507 | - | - | NO | European | Rong et al(2013) |
| SNP | rs1571801 | 9q33 | 123467194 | T | DAB2IP | 1.07 | 0.523 | - | - | NO | European | Rong et al(2013) |
| SNP | rs12418451 | 11q13 | 68691995 | A | - | 0.93 | 0.524 | - | - | NO | European | Rong et al(2013) |
| SNP | rs2055109 | 3p11.2 | 87550022 | C | - | 1.06 | 0.533 | - | - | NO | Japanese | Rong et al(2013) |
| SNP | rs902774 | 12q13 | 51560171 | G | - | 1.18 | 0.536 | - | - | NO | European | Rong et al(2013) |
| SNP | rs2292884 | 2q37.3 | 238107965 | A | MLPH | 1.03 | 0.596 | - | - | NO | European | Rong et al(2013) |
| SNP | rs17021918 | 4q22 | 95781900 | C | PDLIM5 | 1.03 | 0.596 | - | - | NO | European | Rong et al(2013) |
| SNP | rs2735839 | 19q13 | 56056435 | A | KLK3 | 1.02 | 0.718 | - | - | NO | European | Rong et al(2013) |
| SNP | rs5945619 | Xp11 | 51241672 | T | NUDT11 | 1.02 | 0.917 | - | - | NO | European | Rong et al(2013) |
| SNP | rs9623117 | 22q13 | 38782065 | C | TNRC6B | 1.01 | 0.918 | - | - | NO | European | Rong et al(2013) |
| SNP | rs4962416 | 10q26 | 126686862 | G | CTBP2 | - | - | - | - | NO | European | Rong et al(2013) |
| SNP | rs7210100 | 17q21.32 | 44791748 | A | ZNF652 | - | - | - | - | NO | African | Rong et al(2013) |
| SNP | rs5919432 | Xq12 | 66938275 | A | AR | - | - | - | - | NO | European | Rong et al(2013) |
| SNP | rs1815009 | 15 | 99504671 | C | - | 0.7823 | 0.000284 | - | - | - | - | Chen et al(2013) |
| SNP | rs3743250 | 15 | 99505316 | T | - | 0.7815 | 0.000298 | - | - | - | - | Chen et al(2013) |
| SNP | rs3743249 | 15 | 99505423 | T | - | 0.7885 | 0.000435 | - | - | - | - | Chen et al(2013) |
| SNP | rs2517959 | 17 | 37846512 | T | - | 0.7866 | 0.000527 | - | - | - | - | Chen et al(2013) |
| SNP | rs2643194 | 17 | 37853048 | T | - | 0.7668 | 0.00055 | - | - | - | - | Chen et al(2013) |
| SNP | rs2517960 | 17 | 37846521 | C | - | 0.7874 | 0.000552 | - | - | - | - | Chen et al(2013) |
| SNP | rs138246471 | 8 | 142000000 | G | - | 0.2969 | 0.000631 | - | - | - | - | Chen et al(2013) |
| SNP | rs115597780 | 6 | 42007877 | T | - | 0.3681 | 0.000708 | - | - | - | - | Chen et al(2013) |
| SNP | rs149917140 | 6 | 42010175 | T | - | 0.3815 | 0.000733 | - | - | - | - | Chen et al(2013) |
| SNP | rs4331978 | 6 | 42009331 | T | - | 0.3762 | 0.000735 | - | - | - | - | Chen et al(2013) |
| SNP | rs2088126 | 17 | 37879030 | G | - | 0.7697 | 0.000736 | - | - | - | - | Chen et al(2013) |
| SNP | rs903506 | 17 | 37879762 | A | - | 0.7691 | 0.000751 | - | - | - | - | Chen et al(2013) |
| SNP | rs2643195 | 17 | 37853118 | G | - | 0.7736 | 0.000823 | - | - | - | - | Chen et al(2013) |
| SNP | rs4252627 | 17 | 37868715 | T | - | 0.7736 | 0.001023 | - | - | - | - | Chen et al(2013) |
| SNP | rs78790875 | 6 | 42009106 | G | - | 0.3907 | 0.001135 | - | - | - | - | Chen et al(2013) |
| SNP | rs2517951 | 17 | 37853097 | T | - | 0.7788 | 0.001152 | - | - | - | - | Chen et al(2013) |
| SNP | rs2904766 | 17 | 37848677 | G | - | 0.7725 | 0.001196 | - | - | - | - | Chen et al(2013) |
| SNP | rs1810132 | 17 | 37866005 | T | - | 0.7776 | 0.001207 | - | - | - | - | Chen et al(2013) |
| SNP | rs2934971 | 17 | 37854507 | T | - | 0.7805 | 0.00134 | - | - | - | - | Chen et al(2013) |
| SNP | rs2654981 | 15 | 99505129 | C | - | 1.264 | 0.001388 | - | - | - | - | Chen et al(2013) |
| SNP | rs79132119 | 6 | 42000303 | T | - | 0.3667 | 0.001406 | - | - | - | - | Chen et al(2013) |
| SNP | rs61552325 | 17 | 37884037 | G | - | 0.7795 | 0.001465 | - | - | - | - | Chen et al(2013) |
| SNP | rs2934967 | 17 | 37870378 | A | - | 0.7813 | 0.001494 | - | - | - | - | Chen et al(2013) |
| SNP | rs7998124 | 13 | 28917308 | T | - | 0.7341 | 0.001648 | - | - | - | - | Chen et al(2013) |
| SNP | rs2952156 | 17 | 37876835 | G | - | 0.7828 | 0.001667 | - | - | - | - | Chen et al(2013) |
| SNP | rs17172432 | 7 | 55141317 | C | EGFR | 0.673 | 0.001768 | - | - | YES | - | Chen et al(2013) |
| SNP | rs80080085 | 6 | 41999003 | T | - | 0.3765 | 0.002063 | - | - | - | - | Chen et al(2013) |
| SNP | rs1565923 | 17 | 37858678 | G | - | 0.7887 | 0.002165 | - | - | - | - | Chen et al(2013) |
| SNP | rs2952157 | 17 | 37877412 | A | - | 0.7887 | 0.002419 | - | - | - | - | Chen et al(2013) |
| SNP | rs144352608 | 6 | 42001948 | T | - | 0.3961 | 0.002459 | - | - | - | - | Chen et al(2013) |
| SNP | rs191482937 | 11 | 104000000 | G | - | 0.4369 | 0.002512 | - | - | - | - | Chen et al(2013) |
| SNP | rs74916404 | 6 | 42012488 | T | - | 0.4636 | 0.00286 | - | - | - | - | Chen et al(2013) |
| SNP | rs2952155 | 17 | 37861718 | C | - | 0.7943 | 0.002861 | - | - | - | - | Chen et al(2013) |
| SNP | rs11653998 | 17 | 37877447 | G | - | 0.7929 | 0.003031 | - | - | - | - | Chen et al(2013) |
| SNP | rs147804102 | 6 | 42004869 | T | - | 0.3947 | 0.003121 | - | - | - | - | Chen et al(2013) |
| SNP | rs8038415 | 15 | 99499434 | T | - | 1.235 | 0.003128 | - | - | - | - | Chen et al(2013) |
| SNP | rs2016347 | 15 | 99503800 | G | - | 0.8188 | 0.003659 | - | - | - | - | Chen et al(2013) |
| SNP | rs13086363 | 3 | 12675709 | T | - | 0.6767 | 0.003728 | - | - | - | - | Chen et al(2013) |
| SNP | rs8033670 | 15 | 99498879 | T | - | 1.224 | 0.003738 | - | - | - | - | Chen et al(2013) |
| SNP | rs28529403 | 16 | 30134656 | T | - | 0.787 | 0.004563 | - | - | - | - | Chen et al(2013) |
| SNP | rs2684788 | 15 | 99504437 | T | - | 0.8251 | 0.004625 | - | - | - | - | Chen et al(2013) |
| SNP | rs10895290 | 11 | 102000000 | A | - | 0.7987 | 0.004645 | - | - | - | - | Chen et al(2013) |
| SNP | rs148447776 | 6 | 42001134 | A | - | 0.4228 | 0.004997 | - | - | - | - | Chen et al(2013) |
| SNP | rs12634077 | 3 | 12674566 | A | - | 0.6862 | 0.004998 | - | - | - | - | Chen et al(2013) |
| SNP | rs74581689 | 7 | 55102337 | T | - | 0.517 | 0.005207 | - | - | - | - | Chen et al(2013) |
| SNP | rs1391868 | 3 | 12679466 | T | - | 0.6884 | 0.005421 | - | - | - | - | Chen et al(2013) |
| SNP | rs2872060 | 15 | 99499493 | T | - | 1.218 | 0.005563 | - | - | - | - | Chen et al(2013) |
| SNP | rs78815931 | 3 | 12678203 | G | - | 0.6895 | 0.005584 | - | - | - | - | Chen et al(2013) |
| SNP | rs17172434 | 7 | 55144430 | G | EGFR | 0.6899 | 0.005871 | - | - | - | - | Chen et al(2013) |
| SNP | rs147577568 | 6 | 42001911 | A | - | 0.4275 | 0.006007 | - | - | - | - | Chen et al(2013) |
| SNP | rs55916282 | 4 | 55993306 | A | - | 1.352 | 0.006255 | - | - | - | - | Chen et al(2013) |
| SNP | rs17172438 | 7 | 55151537 | C | EGFR | 0.6003 | 0.006644 | - | - | YES | - | Chen et al(2013) |
| SNP | rs4684867 | 3 | 12659494 | G | - | 0.6964 | 0.007133 | - | - | - | - | Chen et al(2013) |
| SNP | rs9672254 | 15 | 99498085 | C | - | 1.207 | 0.007374 | - | - | - | - | Chen et al(2013) |
| SNP | rs622227 | 13 | 29039214 | C | - | 1.532 | 0.008899 | - | - | - | - | Chen et al(2013) |
| SNP | rs5746207 | 3 | 12648156 | G | - | 0.6994 | 0.008937 | - | - | - | - | Chen et al(2013) |
| SNP | rs78968063 | 11 | 69457069 | A | - | 0.04467 | 0.009026 | - | - | - | - | Chen et al(2013) |
| SNP | rs12538371 | 7 | 55236020 | C | - | 0.1536 | 0.009067 | - | - | - | - | Chen et al(2013) |
| SNP | rs142937763 | 6 | 42012786 | T | - | 0.4891 | 0.009335 | - | - | - | - | Chen et al(2013) |
| SNP | rs66740303 | 7 | 55159875 | C | - | 0.5336 | 0.009352 | - | - | - | - | Chen et al(2013) |
| SNP | rs61764202 | 16 | 30134679 | C | - | 0.8097 | 0.009452 | - | - | - | - | Chen et al(2013) |
| SNP | rs3217805 | 12 | 4388084 | G | - | 0.3273 | 0.009554 | - | - | - | - | Chen et al(2013) |
| SNP | rs2904767 | 17 | 37850541 | C | - | 0.6597 | 0.009577 | - | - | - | - | Chen et al(2013) |
| SNP | rs5746194 | 3 | 12658520 | G | - | 0.7044 | 0.00968 | - | - | - | - | Chen et al(2013) |
| SNP | rs13069889 | 3 | 12667680 | T | - | 0.7056 | 0.00992 | - | - | - | - | Chen et al(2013) |
| SNP | rs904464 | 3 | 12642949 | C | - | 0.7108 | 0.01016 | - | - | - | - | Chen et al(2013) |
| SNP | rs56054777 | 2 | 39278250 | G | - | 0.2798 | 0.01023 | - | - | - | - | Chen et al(2013) |
| SNP | rs12629082 | 3 | 12669448 | G | - | 0.7014 | 0.01023 | - | - | - | - | Chen et al(2013) |
| SNP | rs2871866 | 15 | 99221888 | C | - | 0.8198 | 0.01028 | - | - | - | - | Chen et al(2013) |
| SNP | rs140233478 | 4 | 158000000 | T | - | 4.175 | 0.01029 | - | - | - | - | Chen et al(2013) |
| SNP | rs150753022 | 4 | 158000000 | A | - | 4.169 | 0.01036 | - | - | - | - | Chen et al(2013) |
| SNP | rs3025012 | 6 | 43747962 | G | - | 0.3158 | 0.01071 | - | - | - | - | Chen et al(2013) |
| SNP | rs9344 | 11 | 69462910 | G | - | 1.693 | 0.01088 | - | - | - | - | Chen et al(2013) |
| SNP | rs3025018 | 6 | 43748795 | T | - | 0.3419 | 0.01109 | - | - | - | - | Chen et al(2013) |
| SNP | rs6795441 | 3 | 12702562 | A | - | 0.7327 | 0.01131 | - | - | - | - | Chen et al(2013) |
| SNP | rs11569095 | 4 | 111000000 | G | - | 23.92 | 0.01135 | - | - | - | - | Chen et al(2013) |
| SNP | rs187724013 | 4 | 158000000 | G | - | 5.03 | 0.01143 | - | - | - | - | Chen et al(2013) |
| SNP | rs142022576 | 15 | 66713127 | A | - | 2.197 | 0.01145 | - | - | - | - | Chen et al(2013) |
| SNP | rs11713601 | 3 | 12662117 | G | - | 0.7133 | 0.0115 | - | - | - | - | Chen et al(2013) |
| SNP | rs6792773 | 3 | 12668726 | T | - | 0.5746 | 0.01162 | - | - | - | - | Chen et al(2013) |
| SNP | rs145939286 | 8 | 142000000 | A | - | 0.177 | 0.01202 | - | - | - | - | Chen et al(2013) |
| SNP | rs3743251 | 15 | 99504129 | A | - | 0.8425 | 0.01246 | - | - | - | - | Chen et al(2013) |
| SNP | rs7738113 | 6 | 41962692 | T | - | 0.1198 | 0.01276 | - | - | - | - | Chen et al(2013) |
| SNP | rs2286962 | 7 | 55114093 | C | - | 0.6094 | 0.01283 | - | - | - | - | Chen et al(2013) |
| SNP | rs73130368 | 3 | 12667374 | T | - | 0.5795 | 0.0132 | - | - | - | - | Chen et al(2013) |
| SNP | rs7995976 | 13 | 28941060 | A | - | 0.8381 | 0.01375 | - | - | - | - | Chen et al(2013) |
| SNP | rs2348199 | 3 | 12691265 | G | - | 0.7161 | 0.01388 | - | - | - | - | Chen et al(2013) |
| SNP | rs10438491 | 15 | 99489265 | T | - | 0.8484 | 0.01391 | - | - | - | - | Chen et al(2013) |
| SNP | rs2596830 | 3 | 12631774 | G | - | 0.7218 | 0.01431 | - | - | - | - | Chen et al(2013) |
| SNP | rs9852359 | 3 | 12693430 | T | - | 0.5829 | 0.01464 | - | - | - | - | Chen et al(2013) |
| SNP | rs4684871 | 3 | 12697498 | G | - | 0.7272 | 0.01512 | - | - | - | - | Chen et al(2013) |
| SNP | rs10460973 | 3 | 12704163 | C | - | 0.7395 | 0.01588 | - | - | - | - | Chen et al(2013) |
| SNP | rs904453 | 3 | 12704894 | G | - | 0.7395 | 0.01588 | - | - | - | - | Chen et al(2013) |
| SNP | rs111260399 | 4 | 111000000 | G | - | 0.3942 | 0.016 | - | - | - | - | Chen et al(2013) |
| SNP | rs625980 | 13 | 29038388 | T | - | 1.495 | 0.01641 | - | - | - | - | Chen et al(2013) |
| SNP | rs9809947 | 3 | 12655944 | C | - | 0.5748 | 0.01672 | - | - | - | - | Chen et al(2013) |
| SNP | rs2016492 | 3 | 12634518 | C | - | 0.74 | 0.01703 | - | - | - | - | Chen et al(2013) |
| SNP | rs56728557 | 13 | 29029431 | A | - | 1.495 | 0.01704 | - | - | - | - | Chen et al(2013) |
| SNP | rs5746182 | 3 | 12672550 | G | - | 0.6003 | 0.01726 | - | - | - | - | Chen et al(2013) |
| SNP | rs57884385 | 3 | 12673551 | C | - | 0.6003 | 0.01726 | - | - | - | - | Chen et al(2013) |
| SNP | rs74325933 | 15 | 99321452 | C | - | 0.7383 | 0.01751 | - | - | - | - | Chen et al(2013) |
| SNP | rs77707425 | 3 | 12675810 | C | - | 0.5988 | 0.01764 | - | - | - | - | Chen et al(2013) |
| SNP | rs6491275 | 13 | 28941930 | A | - | 0.8437 | 0.01775 | - | - | - | - | Chen et al(2013) |
| SNP | rs111654948 | 3 | 12677599 | A | - | 0.5332 | 0.0181 | - | - | - | - | Chen et al(2013) |
| SNP | rs11249739 | 5 | 180000000 | T | - | 0.8036 | 0.01817 | - | - | - | - | Chen et al(2013) |
| SNP | rs1858830 | 7 | 116000000 | C | - | 1.261 | 0.01836 | - | - | - | - | Chen et al(2013) |
| SNP | rs145099651 | 18 | 60836658 | T | - | 7.706 | 0.01846 | - | - | - | - | Chen et al(2013) |
| SNP | rs729969 | 7 | 55128207 | A | - | 0.6379 | 0.01849 | - | - | - | - | Chen et al(2013) |
| SNP | rs11773818 | 7 | 55123968 | C | EGFR | 0.8013 | 0.01856 | - | - | YES | - | Chen et al(2013) |
| SNP | rs2454437 | 3 | 12638730 | A | - | 0.7343 | 0.01862 | - | - | - | - | Chen et al(2013) |
| SNP | rs4234513 | 3 | 12703063 | G | - | 0.7452 | 0.01883 | - | - | - | - | Chen et al(2013) |
| SNP | rs1800477 | 18 | 60985773 | T | - | 0.4166 | 0.01925 | - | - | - | - | Chen et al(2013) |
| SNP | rs143393162 | 7 | 55105896 | A | - | 2.803 | 0.01943 | - | - | - | - | Chen et al(2013) |
| SNP | rs11709504 | 3 | 12674199 | C | - | 0.6081 | 0.01973 | - | - | - | - | Chen et al(2013) |
| SNP | rs35006544 | 5 | 180000000 | T | - | 0.7484 | 0.01974 | - | - | - | - | Chen et al(2013) |
| SNP | rs2283051 | 7 | 116000000 | C | - | 0.3053 | 0.0199 | - | - | - | - | Chen et al(2013) |
| SNP | rs9849807 | 3 | 12675026 | G | - | 0.6067 | 0.02017 | - | - | - | - | Chen et al(2013) |
| SNP | rs9657438 | 8 | 142000000 | T | - | 0.1786 | 0.02037 | - | - | - | - | Chen et al(2013) |
| SNP | rs12669701 | 7 | 55131670 | A | - | 0.7831 | 0.02051 | - | - | - | - | Chen et al(2013) |
| SNP | rs3735064 | 7 | 55144833 | C | EGFR | 0.7874 | 0.02077 | - | - | - | - | Chen et al(2013) |
| SNP | rs150708329 | 12 | 103000000 | G | - | 0.2639 | 0.02155 | - | - | - | - | Chen et al(2013) |
| SNP | rs111962445 | 3 | 12695689 | A | - | 0.6014 | 0.02194 | - | - | - | - | Chen et al(2013) |
| SNP | rs144522369 | 4 | 158000000 | C | - | 0.2486 | 0.02202 | - | - | - | - | Chen et al(2013) |
| SNP | rs2442807 | 3 | 12629965 | C | - | 0.7403 | 0.02218 | - | - | - | - | Chen et al(2013) |
| SNP | rs7317419 | 13 | 29025559 | T | - | 1.481 | 0.02223 | - | - | - | - | Chen et al(2013) |
| SNP | rs10507386 | 13 | 29028554 | T | - | 1.47 | 0.02248 | - | - | - | - | Chen et al(2013) |
| SNP | rs1398873 | 15 | 99396105 | T | - | 0.6099 | 0.02262 | - | - | - | - | Chen et al(2013) |
| SNP | rs881365 | 11 | 104000000 | T | - | 0.7121 | 0.02276 | - | - | - | - | Chen et al(2013) |
| SNP | rs9817675 | 3 | 12676113 | T | - | 0.6146 | 0.023 | - | - | - | - | Chen et al(2013) |
| SNP | rs7162314 | 15 | 99489916 | G | - | 0.8548 | 0.02309 | - | - | - | - | Chen et al(2013) |
| SNP | rs3794402 | 13 | 28924832 | C | - | 2.008 | 0.02338 | - | - | - | - | Chen et al(2013) |
| SNP | rs8034284 | 15 | 99496248 | C | - | 1.175 | 0.02342 | - | - | - | - | Chen et al(2013) |
| SNP | rs76469750 | 8 | 142000000 | T | - | 0.538 | 0.02345 | - | - | - | - | Chen et al(2013) |
| SNP | rs10071147 | 5 | 180000000 | A | - | 1.183 | 0.02353 | - | - | - | - | Chen et al(2013) |
| SNP | rs619031 | 13 | 29039871 | T | - | 1.504 | 0.02367 | - | - | - | - | Chen et al(2013) |
| SNP | rs28438589 | 8 | 142000000 | C | - | 0.7181 | 0.02372 | - | - | - | - | Chen et al(2013) |
| SNP | rs7637392 | 3 | 12696433 | G | - | 0.6102 | 0.02379 | - | - | - | - | Chen et al(2013) |
| SNP | rs11238349 | 7 | 55156071 | G | EGFR | 0.766 | 0.02387 | - | - | YES | - | Chen et al(2013) |
| SNP | rs143510792 | 8 | 142000000 | T | - | 0.2692 | 0.02427 | - | - | - | - | Chen et al(2013) |
| SNP | rs963959 | 3 | 12649937 | T | - | 0.7277 | 0.0244 | - | - | - | - | Chen et al(2013) |
| SNP | rs79234561 | 5 | 180000000 | A | - | 0.1429 | 0.02451 | - | - | - | - | Chen et al(2013) |
| SNP | rs9508017 | 13 | 28934364 | T | - | 0.8497 | 0.02472 | - | - | - | - | Chen et al(2013) |
| SNP | rs6978771 | 7 | 55140296 | C | EGFR | 0.793 | 0.02475 | - | - | - | - | Chen et al(2013) |
| SNP | rs59821954 | 3 | 12687613 | C | - | 0.6127 | 0.02495 | - | - | - | - | Chen et al(2013) |
| SNP | rs145732636 | 15 | 99452581 | T | - | 0.5924 | 0.02541 | - | - | - | - | Chen et al(2013) |
| SNP | rs984654 | 7 | 55144156 | T | EGFR | 0.8001 | 0.02558 | - | - | YES | - | Chen et al(2013) |
| SNP | rs11865086 | 16 | 30130493 | C | - | 0.855 | 0.02561 | - | - | - | - | Chen et al(2013) |
| SNP | rs11923427 | 3 | 12663835 | G | - | 0.611 | 0.02563 | - | - | - | - | Chen et al(2013) |
| SNP | rs3773345 | 3 | 12642945 | C | - | 0.6073 | 0.02582 | - | - | - | - | Chen et al(2013) |
| SNP | rs6490306 | 13 | 29025304 | A | - | 1.47 | 0.02599 | - | - | - | - | Chen et al(2013) |
| SNP | rs56363132 | 3 | 12680403 | C | - | 0.6145 | 0.02602 | - | - | - | - | Chen et al(2013) |
| SNP | rs9837418 | 3 | 12685012 | A | - | 0.6145 | 0.02602 | - | - | - | - | Chen et al(2013) |
| SNP | rs75546857 | 7 | 55114721 | G | - | 0.6472 | 0.02622 | - | - | - | - | Chen et al(2013) |
| SNP | rs6958497 | 7 | 55161746 | C | EGFR | 2.64 | 0.02663 | - | - | - | - | Chen et al(2013) |
| SNP | rs4385869 | 11 | 102000000 | A | - | 0.7975 | 0.02664 | - | - | - | - | Chen et al(2013) |
| SNP | rs9864781 | 3 | 12683446 | T | - | 0.5925 | 0.02669 | - | - | - | - | Chen et al(2013) |
| SNP | rs38846 | 7 | 116000000 | C | - | 0.4244 | 0.02683 | - | - | - | - | Chen et al(2013) |
| SNP | rs2302535 | 7 | 55154688 | A | EGFR | 0.7708 | 0.02693 | - | - | - | - | Chen et al(2013) |
| SNP | rs13165395 | 5 | 180000000 | C | - | 1.269 | 0.02702 | - | - | - | - | Chen et al(2013) |
| SNP | rs3209052 | 13 | 28874695 | C | - | 0.8146 | 0.02707 | - | - | - | - | Chen et al(2013) |
| SNP | rs76427221 | 3 | 12646751 | A | - | 0.5427 | 0.02717 | - | - | - | - | Chen et al(2013) |
| SNP | rs7982257 | 13 | 28921481 | G | - | 0.8451 | 0.02721 | - | - | - | - | Chen et al(2013) |
| SNP | rs3776413 | 5 | 180000000 | T | - | 0.7976 | 0.02723 | - | - | - | - | Chen et al(2013) |
| SNP | rs1874942 | 3 | 12690343 | G | - | 0.7407 | 0.02751 | - | - | - | - | Chen et al(2013) |
| SNP | rs58518391 | 13 | 29031060 | A | - | 1.454 | 0.02784 | - | - | - | - | Chen et al(2013) |
| SNP | rs58759217 | 13 | 29030978 | T | - | 1.454 | 0.0279 | - | - | - | - | Chen et al(2013) |
| SNP | rs3025015 | 6 | 43748350 | A | - | 0.4302 | 0.02805 | - | - | - | - | Chen et al(2013) |
| SNP | rs183883220 | 11 | 104000000 | A | - | 0.1451 | 0.02843 | - | - | - | - | Chen et al(2013) |
| SNP | rs149330409 | 11 | 104000000 | T | - | 0.1451 | 0.02843 | - | - | - | - | Chen et al(2013) |
| SNP | rs115391410 | 7 | 55113796 | C | - | 0.6422 | 0.02887 | - | - | - | - | Chen et al(2013) |
| SNP | rs11977660 | 7 | 55162336 | T | - | 0.8577 | 0.02899 | - | - | - | - | Chen et al(2013) |
| SNP | rs79868943 | 7 | 55159109 | A | - | 0.6313 | 0.02911 | - | - | - | - | Chen et al(2013) |
| SNP | rs34867831 | 13 | 29022711 | C | - | 1.502 | 0.02915 | - | - | - | - | Chen et al(2013) |
| SNP | rs2329881 | 22 | 22170312 | T | - | 0.1459 | 0.02943 | - | - | - | - | Chen et al(2013) |
| SNP | rs9875104 | 3 | 12685243 | T | - | 0.5643 | 0.02991 | - | - | - | - | Chen et al(2013) |
| SNP | rs2312491 | 15 | 99508180 | A | - | 0.8474 | 0.02994 | - | - | - | - | Chen et al(2013) |
| SNP | rs17626553 | 13 | 28968770 | C | - | 1.908 | 0.02998 | - | - | - | - | Chen et al(2013) |
| SNP | rs45578135 | 15 | 99485516 | C | - | 0.598 | 0.03002 | - | - | - | - | Chen et al(2013) |
| SNP | rs17336017 | 7 | 55152680 | A | - | 0.638 | 0.0305 | - | - | - | - | Chen et al(2013) |
| SNP | rs3735063 | 7 | 55153164 | C | EGFR | 0.638 | 0.0305 | - | - | - | - | Chen et al(2013) |
| SNP | rs77738372 | 5 | 180000000 | T | - | 0.1865 | 0.03052 | - | - | - | - | Chen et al(2013) |
| SNP | rs77616576 | 13 | 29041593 | T | - | 0.2186 | 0.03062 | - | - | - | - | Chen et al(2013) |
| SNP | rs6001509 | 22 | 39624900 | T | - | 0.6336 | 0.03066 | - | - | - | - | Chen et al(2013) |
| SNP | rs2298989 | 4 | 111000000 | T | - | 1.167 | 0.03084 | - | - | - | - | Chen et al(2013) |
| SNP | rs9513085 | 13 | 28920923 | C | - | 0.8484 | 0.03103 | - | - | - | - | Chen et al(2013) |
| SNP | rs12591122 | 15 | 99498412 | C | - | 0.8608 | 0.03119 | - | - | - | - | Chen et al(2013) |
| SNP | rs2093821 | 13 | 28969209 | C | - | 1.753 | 0.03121 | - | - | - | - | Chen et al(2013) |
| SNP | rs75144654 | 7 | 551646 | A | - | 0.1562 | 0.03143 | - | - | - | - | Chen et al(2013) |
| SNP | rs118137198 | 13 | 29023288 | C | - | 1.498 | 0.03153 | - | - | - | - | Chen et al(2013) |
| SNP | rs3800827 | 7 | 55142654 | A | EGFR | 0.801 | 0.03158 | - | - | - | - | Chen et al(2013) |
| SNP | rs80022884 | 8 | 142000000 | T | - | 0.2158 | 0.03197 | - | - | - | - | Chen et al(2013) |
| SNP | rs2904768 | 17 | 37850571 | T | - | 0.8151 | 0.03197 | - | - | - | - | Chen et al(2013) |
| SNP | rs899169 | 5 | 180000000 | A | - | 0.8619 | 0.03215 | - | - | - | - | Chen et al(2013) |
| SNP | rs9642564 | 7 | 55145334 | G | EGFR | 0.8008 | 0.03226 | - | - | - | - | Chen et al(2013) |
| SNP | rs17537653 | 13 | 28968510 | A | - | 1.894 | 0.03226 | - | - | - | - | Chen et al(2013) |
| SNP | rs75133077 | 3 | 12688513 | T | - | 0.6249 | 0.03238 | - | - | - | - | Chen et al(2013) |
| SNP | rs55762590 | 3 | 12689451 | T | - | 0.6249 | 0.03238 | - | - | - | - | Chen et al(2013) |
| SNP | rs6766666 | 3 | 12690855 | T | - | 0.6249 | 0.03238 | - | - | - | - | Chen et al(2013) |
| SNP | rs150558261 | 8 | 142000000 | C | - | 0.2169 | 0.03238 | - | - | - | - | Chen et al(2013) |
| SNP | rs7542 | 16 | 30125840 | G | - | 0.8544 | 0.03239 | - | - | - | - | Chen et al(2013) |
| SNP | rs79698813 | 7 | 55135897 | G | - | 0.6496 | 0.0324 | - | - | - | - | Chen et al(2013) |
| SNP | rs12437796 | 15 | 99493653 | A | - | 0.8615 | 0.03242 | - | - | - | - | Chen et al(2013) |
| SNP | rs35115159 | 15 | 99490912 | G | - | 0.8625 | 0.03253 | - | - | - | - | Chen et al(2013) |
| SNP | rs12592205 | 15 | 99490596 | T | - | 0.8627 | 0.03266 | - | - | - | - | Chen et al(2013) |
| SNP | rs2256849 | 13 | 29045852 | G | - | 1.45 | 0.03269 | - | - | - | - | Chen et al(2013) |
| SNP | rs5745765 | 7 | 81332213 | C | - | 0.5199 | 0.03317 | - | - | - | - | Chen et al(2013) |
| SNP | rs148833685 | 4 | 111000000 | A | - | 0.4456 | 0.03322 | - | - | - | - | Chen et al(2013) |
| SNP | rs34971240 | 18 | 60923936 | G | - | 0.5595 | 0.03328 | - | - | - | - | Chen et al(2013) |
| SNP | rs11766798 | 7 | 55124319 | G | EGFR | 0.8016 | 0.0333 | - | - | - | - | Chen et al(2013) |
| SNP | rs7636754 | 3 | 12678725 | G | - | 0.6309 | 0.03356 | - | - | - | - | Chen et al(2013) |
| SNP | rs6784435 | 3 | 12682089 | T | - | 0.6309 | 0.03356 | - | - | - | - | Chen et al(2013) |
| SNP | rs5746173 | 3 | 12686353 | G | - | 0.6309 | 0.03356 | - | - | - | - | Chen et al(2013) |
| SNP | rs76161861 | 7 | 81398644 | A | - | 0.4885 | 0.03359 | - | - | - | - | Chen et al(2013) |
| SNP | rs117940891 | 11 | 104000000 | C | - | 0.6772 | 0.03388 | - | - | - | - | Chen et al(2013) |
| SNP | rs141568990 | 11 | 104000000 | G | - | 0.6772 | 0.03388 | - | - | - | - | Chen et al(2013) |
| SNP | rs73132351 | 3 | 12688284 | G | - | 0.6315 | 0.034 | - | - | - | - | Chen et al(2013) |
| SNP | rs5746191 | 3 | 12658774 | A | - | 0.6304 | 0.03411 | - | - | - | - | Chen et al(2013) |
| SNP | rs8001882 | 13 | 28921281 | A | - | 0.8513 | 0.03442 | - | - | - | - | Chen et al(2013) |
| SNP | rs183801378 | 7 | 55154562 | A | - | 10.71 | 0.03443 | - | - | - | - | Chen et al(2013) |
| SNP | rs6778959 | 3 | 12690527 | C | - | 0.6323 | 0.03448 | - | - | - | - | Chen et al(2013) |
| SNP | rs34895288 | 15 | 99496707 | T | - | 0.8659 | 0.03463 | - | - | - | - | Chen et al(2013) |
| SNP | rs12535578 | 7 | 55154586 | G | EGFR | 0.7809 | 0.03486 | - | - | - | - | Chen et al(2013) |
| SNP | rs6784168 | 3 | 12702847 | G | - | 0.6663 | 0.03504 | - | - | - | - | Chen et al(2013) |
| SNP | rs114465516 | 7 | 55150359 | A | - | 0.6467 | 0.03526 | - | - | - | - | Chen et al(2013) |
| SNP | rs2684792 | 15 | 99488081 | A | - | 1.159 | 0.03528 | - | - | - | - | Chen et al(2013) |
| SNP | rs11149523 | 13 | 28995630 | A | - | 0.8404 | 0.03582 | - | - | - | - | Chen et al(2013) |
| SNP | rs9823121 | 3 | 12707277 | T | - | 0.6642 | 0.03584 | - | - | - | - | Chen et al(2013) |
| SNP | rs187262191 | 4 | 111000000 | A | - | 0.3693 | 0.03597 | - | - | - | - | Chen et al(2013) |
| SNP | rs9513087 | 13 | 28928688 | T | - | 0.8582 | 0.03606 | - | - | - | - | Chen et al(2013) |
| SNP | rs11226091 | 11 | 104000000 | C | - | 1.159 | 0.0362 | - | - | - | - | Chen et al(2013) |
| SNP | rs9657467 | 8 | 142000000 | C | - | 0.1523 | 0.03638 | - | - | - | - | Chen et al(2013) |
| SNP | rs9878151 | 3 | 12703714 | C | - | 0.674 | 0.03653 | - | - | - | - | Chen et al(2013) |
| SNP | rs34465640 | 13 | 28998799 | G | - | 0.8424 | 0.03753 | - | - | - | - | Chen et al(2013) |
| SNP | rs139273345 | 6 | 112000000 | T | - | 0.3899 | 0.03796 | - | - | - | - | Chen et al(2013) |
| SNP | rs139234015 | 6 | 112000000 | A | - | 0.3905 | 0.0382 | - | - | - | - | Chen et al(2013) |
| SNP | rs61160416 | 7 | 55128285 | C | - | 0.671 | 0.03822 | - | - | - | - | Chen et al(2013) |
| SNP | rs17086708 | 13 | 29040660 | G | - | 1.463 | 0.03831 | - | - | - | - | Chen et al(2013) |
| SNP | rs34945396 | 4 | 55982784 | C | - | 0.7474 | 0.03833 | - | - | - | - | Chen et al(2013) |
| SNP | rs12429309 | 13 | 28879332 | C | - | 1.163 | 0.03841 | - | - | - | - | Chen et al(2013) |
| SNP | rs4987716 | 18 | 60960979 | A | - | 0.2946 | 0.03843 | - | - | - | - | Chen et al(2013) |
| SNP | rs17335891 | 7 | 55131064 | C | - | 0.8052 | 0.03888 | - | - | - | - | Chen et al(2013) |
| SNP | rs11618844 | 13 | 29034991 | C | - | 1.423 | 0.03893 | - | - | - | - | Chen et al(2013) |
| SNP | rs72818977 | 5 | 180000000 | A | - | 1.323 | 0.039 | - | - | - | - | Chen et al(2013) |
| SNP | rs73489562 | 7 | 140000000 | T | - | 0.7779 | 0.03903 | - | - | - | - | Chen et al(2013) |
| SNP | rs146113622 | 17 | 73351811 | C | - | 0.08258 | 0.03908 | - | - | - | - | Chen et al(2013) |
| SNP | rs78419250 | 6 | 41977713 | G | - | 0.1525 | 0.03927 | - | - | - | - | Chen et al(2013) |
| SNP | rs1558543 | 7 | 55135512 | T | - | 0.658 | 0.03941 | - | - | - | - | Chen et al(2013) |
| SNP | rs28577882 | 7 | 140000000 | A | - | 0.7783 | 0.03946 | - | - | - | - | Chen et al(2013) |
| SNP | rs10242534 | 7 | 140000000 | C | - | 0.7783 | 0.03946 | - | - | - | - | Chen et al(2013) |
| SNP | rs78795510 | 5 | 180000000 | G | - | 0.1608 | 0.03953 | - | - | - | - | Chen et al(2013) |
| SNP | rs5746214 | 3 | 12644753 | G | - | 0.6341 | 0.03955 | - | - | - | - | Chen et al(2013) |
| SNP | rs12437963 | 15 | 99496859 | G | - | 0.8685 | 0.03958 | - | - | - | - | Chen et al(2013) |
| SNP | rs143507850 | 4 | 111000000 | C | - | 0.3219 | 0.03999 | - | - | - | - | Chen et al(2013) |
| SNP | rs149017693 | 11 | 104000000 | C | - | 0.156 | 0.04013 | - | - | - | - | Chen et al(2013) |
| SNP | rs116935757 | 8 | 142000000 | A | - | 0.3636 | 0.04022 | - | - | - | - | Chen et al(2013) |
| SNP | rs671422 | 13 | 29049430 | G | - | 1.435 | 0.04031 | - | - | - | - | Chen et al(2013) |
| SNP | rs55977822 | 3 | 12704438 | A | - | 0.6704 | 0.04041 | - | - | - | - | Chen et al(2013) |
| SNP | rs117656018 | 4 | 111000000 | T | - | 0.323 | 0.04054 | - | - | - | - | Chen et al(2013) |
| SNP | rs111599093 | 7 | 55125897 | A | - | 0.6739 | 0.04054 | - | - | - | - | Chen et al(2013) |
| SNP | rs112317270 | 7 | 55126160 | C | - | 0.6739 | 0.04054 | - | - | - | - | Chen et al(2013) |
| SNP | rs9551471 | 13 | 29007730 | G | - | 0.8462 | 0.04058 | - | - | - | - | Chen et al(2013) |
| SNP | rs2217805 | 17 | 73334164 | C | - | 1.697 | 0.04062 | - | - | - | - | Chen et al(2013) |
| SNP | rs12651220 | 4 | 111000000 | A | - | 0.3235 | 0.04091 | - | - | - | - | Chen et al(2013) |
| SNP | rs13060685 | 3 | 12659392 | G | - | 0.6984 | 0.041 | - | - | - | - | Chen et al(2013) |
| SNP | rs73131843 | 7 | 55117116 | G | - | 0.6929 | 0.04137 | - | - | - | - | Chen et al(2013) |
| SNP | rs10507385 | 13 | 28996960 | C | - | 0.8456 | 0.04146 | - | - | - | - | Chen et al(2013) |
| SNP | rs662560 | 13 | 29067336 | C | - | 1.335 | 0.04182 | - | - | - | - | Chen et al(2013) |
| SNP | rs646347 | 13 | 29044015 | C | - | 1.422 | 0.04189 | - | - | - | - | Chen et al(2013) |
| SNP | rs638889 | 13 | 29044352 | C | - | 1.422 | 0.04199 | - | - | - | - | Chen et al(2013) |
| SNP | rs77374137 | 13 | 29030190 | T | - | 1.461 | 0.04204 | - | - | - | - | Chen et al(2013) |
| SNP | rs142947254 | 8 | 142000000 | G | - | 0.2653 | 0.0422 | - | - | - | - | Chen et al(2013) |
| SNP | rs12821878 | 12 | 103000000 | A | - | 1.388 | 0.04228 | - | - | - | - | Chen et al(2013) |
| SNP | rs140492735 | 5 | 180000000 | T | - | 1.318 | 0.04245 | - | - | - | - | Chen et al(2013) |
| SNP | rs11569100 | 4 | 111000000 | C | - | 0.739 | 0.04249 | - | - | - | - | Chen et al(2013) |
| SNP | rs73455407 | 13 | 29023682 | G | - | 1.462 | 0.04249 | - | - | - | - | Chen et al(2013) |
| SNP | rs79163032 | 4 | 55987777 | A | - | 1.87 | 0.04251 | - | - | - | - | Chen et al(2013) |
| SNP | rs17086739 | 13 | 29051717 | A | - | 1.408 | 0.04288 | - | - | - | - | Chen et al(2013) |
| SNP | rs117968512 | 13 | 29047362 | A | - | 1.455 | 0.04302 | - | - | - | - | Chen et al(2013) |
| SNP | rs6777175 | 3 | 12646255 | T | - | 0.64 | 0.04305 | - | - | - | - | Chen et al(2013) |
| SNP | rs6442319 | 3 | 12647029 | A | - | 0.64 | 0.04305 | - | - | - | - | Chen et al(2013) |
| SNP | rs2290162 | 3 | 12647457 | G | - | 0.64 | 0.04305 | - | - | - | - | Chen et al(2013) |
| SNP | rs5746206 | 3 | 12648255 | A | - | 0.64 | 0.04305 | - | - | - | - | Chen et al(2013) |
| SNP | rs3773349 | 3 | 12649732 | C | - | 0.64 | 0.04305 | - | - | - | - | Chen et al(2013) |
| SNP | rs72754807 | 15 | 99506740 | A | - | 1.875 | 0.04318 | - | - | - | - | Chen et al(2013) |
| SNP | rs75726430 | 7 | 55148154 | G | - | 0.6542 | 0.04344 | - | - | - | - | Chen et al(2013) |
| SNP | rs9554330 | 13 | 29003444 | A | - | 0.8501 | 0.04377 | - | - | - | - | Chen et al(2013) |
| SNP | rs17172436 | 7 | 55148715 | C | EGFR | 0.6598 | 0.04405 | - | - | - | - | Chen et al(2013) |
| SNP | rs72752895 | 15 | 99497467 | T | - | 0.8694 | 0.04406 | - | - | - | - | Chen et al(2013) |
| SNP | rs11226075 | 11 | 104000000 | C | - | 0.8655 | 0.04411 | - | - | - | - | Chen et al(2013) |
| SNP | rs9513070 | 13 | 28879839 | G | - | 1.162 | 0.04426 | - | - | - | - | Chen et al(2013) |
| SNP | rs57132241 | 7 | 116000000 | A | - | 1.392 | 0.04477 | - | - | - | - | Chen et al(2013) |
| SNP | rs55717377 | 17 | 37850569 | T | - | 0.8181 | 0.0449 | - | - | - | - | Chen et al(2013) |
| SNP | rs13070228 | 3 | 12706914 | A | - | 0.685 | 0.04497 | - | - | - | - | Chen et al(2013) |
| SNP | rs659413 | 13 | 29043435 | C | - | 1.424 | 0.04522 | - | - | - | - | Chen et al(2013) |
| SNP | rs77605019 | 15 | 99387367 | G | - | 1.634 | 0.04564 | - | - | - | - | Chen et al(2013) |
| SNP | rs151262481 | 4 | 158000000 | C | - | 4.822 | 0.04577 | - | - | - | - | Chen et al(2013) |
| SNP | rs9991904 | 4 | 111000000 | G | - | 0.7431 | 0.04616 | - | - | - | - | Chen et al(2013) |
| SNP | rs74785122 | 15 | 99378882 | A | - | 1.632 | 0.0462 | - | - | - | - | Chen et al(2013) |
| SNP | rs17289533 | 7 | 55204109 | A | - | 0.2141 | 0.04625 | - | - | - | - | Chen et al(2013) |
| SNP | rs3797102 | 5 | 180000000 | G | - | 1.149 | 0.04646 | - | - | - | - | Chen et al(2013) |
| SNP | rs78602116 | 13 | 29028661 | A | - | 1.445 | 0.0465 | - | - | - | - | Chen et al(2013) |
| SNP | rs76839733 | 13 | 29028682 | G | - | 1.445 | 0.0465 | - | - | - | - | Chen et al(2013) |
| SNP | rs3735062 | 7 | 55153237 | A | EGFR | 0.6426 | 0.04664 | - | - | - | - | Chen et al(2013) |
| SNP | rs7709359 | 5 | 180000000 | G | - | 1.163 | 0.04699 | - | - | - | - | Chen et al(2013) |
| SNP | rs5746187 | 3 | 12659419 | G | - | 0.6494 | 0.04704 | - | - | - | - | Chen et al(2013) |
| SNP | rs10488140 | 7 | 55138388 | T | EGFR | 0.7337 | 0.04782 | - | - | - | - | Chen et al(2013) |
| SNP | rs151015237 | 7 | 81389727 | C | - | 0.06838 | 0.0479 | - | - | - | - | Chen et al(2013) |
| SNP | rs74032119 | 15 | 99488362 | A | - | 0.5219 | 0.04816 | - | - | - | - | Chen et al(2013) |
| SNP | rs114962406 | 15 | 99488579 | A | - | 0.5219 | 0.04816 | - | - | - | - | Chen et al(2013) |
| SNP | rs11618340 | 13 | 29066152 | C | - | 1.324 | 0.04845 | - | - | - | - | Chen et al(2013) |
| SNP | rs7242542 | 18 | 60901926 | T | - | 1.206 | 0.04853 | - | - | - | - | Chen et al(2013) |
| SNP | rs939626 | 15 | 99493176 | C | - | 1.171 | 0.04906 | - | - | - | - | Chen et al(2013) |
| SNP | rs145951686 | 2 | 39223403 | T | - | 0.3992 | 0.04919 | - | - | - | - | Chen et al(2013) |
| SNP | rs6464036 | 7 | 140000000 | T | - | 0.79 | 0.04934 | - | - | - | - | Chen et al(2013) |
| SNP | rs917880 | 7 | 55162011 | T | - | 0.8416 | 0.04969 | - | - | - | - | Chen et al(2013) |
| SNP | rs6840890 | 4 | 111000000 | C | - | 0.4094 | 0.04974 | - | - | - | - | Chen et al(2013) |
| SNP | rs140001952 | 4 | 111000000 | C | - | 0.4095 | 0.04981 | - | - | - | - | Chen et al(2013) |
| CNV | - | 19 | 20079177-21077133 | - | *CR593334,CR614976,LOC284441,ZNF430,ZNF486 | 12.6 | 2.39×10^-3^ | Del | risk | potential | - | Wu et al(2018) |
| CNV | - | 11 | 67258202-67488532 | - | AK091996,BC110365,CR602694 | 2.08 | 9.22×10^-5^ | Del | risk | potential | - | Wu et al(2018) |
| CNV | - | 10 | 59242376-59438056 | - | intergenic | 6.3 | 9.08×10**^-7^** | Del | risk | identified | - | Wu et al(2018) |
| CNV | - | 21 | 20315468-20449022 | - | intergenic | INF | 9.08×10**^-7^** | Del | risk | identified | - | Wu et al(2018) |
| CNV | - | 11 | 88204734-88263638 | - | GRM5 | INF | 8.96×10**^-54^** | Del | risk | identified | - | Wu et al(2018) |
| CNV | - | 16 | 2136084-2187363 | - | CASKIN1,RAB26,SNORD60,TRAF7 | INF | 8.23×10**^-7^** | Del | risk | identified | - | Wu et al(2018) |
| CNV | - | 16 | 616990-718025 | - | *AK128777,AL360260,C16orf13,C16orf14,CCDC78 | INF | 8.19×10^-4^ | Del | risk | potential | - | Wu et al(2018) |
| CNV | - | 3 | 4132503-4187007 | - | SUMF1 | 4.89 | 7.86×10**^-32^** | Del | risk | identified | - | Wu et al(2018) |
| CNV | - | 6 | 79031809-79086086 | - | intergenic | 7.02 | 6.77×10^-3^ | Dup | risk | potential | - | Wu et al(2018) |
| CNV | - | 11 | 88336310-88384073 | - | GRM5 | 13.72 | 6.63×10^-5^ | Dup | risk | potential | - | Wu et al(2018) |
| CNV | - | 18 | 14737958-14828909 | - | ANKRD30B | 3.74 | 6.25×10^-3^ | Dup | risk | potential | - | Wu et al(2018) |
| CNV | - | 6 | 32601768-32664508 | - | AK293020,HLA-DRB1,HLA-DRB5 | 8.9 | 5.28×10^-3^ | Dup | risk | potential | - | Wu et al(2018) |
| CNV | - | 10 | 22249227-22376157 | - | DNAJC1 | INF | 5.28×10^-3^ | Dup | risk | potential | - | Wu et al(2018) |
| CNV | - | 15 | 19257404-19408394 | - | A26B1,DQ582260,M84131,POTEB | INF | 4.64×10^-2^ | Dup | risk | potential | - | Wu et al(2018) |
| CNV | - | 6 | 80285260-80386902 | - | LCA5 | INF | 4.59×10^-3^ | Dup | risk | potential | - | Wu et al(2018) |
| CNV | - | 4 | 9003188-9169160 | - | DEFB131,LOC650293 | 5.13 | 3.98×10^-2^ | Dup | risk | potential | - | Wu et al(2018) |
| CNV | - | 5 | 109845447-109992561 | - | FLJ43080 | 9.79 | 3.98×10^-2^ | Dup | risk | potential | - | Wu et al(2018) |
| CNV | - | 5 | 32143138-32144121 | - | KIAA0300,PDZD2 | 2.04 | 3.98×10^-2^ | Dup | risk | potential | - | Wu et al(2018) |
| CNV | - | 13 | 63130342-63185384 | - | intergenic | INF | 3.47×10^-5^ | Dup | risk | potential | - | Wu et al(2018) |
| CNV | - | 16 | 32073040-32556470 | - | BC041879,BC042588,DQ571479,TP53TG3b,X69637 | INF | 3.31×10^-2^ | Dup | risk | potential | - | Wu et al(2018) |
| CNV | - | 11 | 58563817-58634485 | - | AX746988,FAM111B | 2.22 | 3.20×10^-2^ | Dup | risk | potential | - | Wu et al(2018) |
| CNV | - | 12 | 85219842-85281435 | - | MGAT4C | INF | 3.13×10^-3^ | Dup | risk | potential | - | Wu et al(2018) |
| CNV | - | 18 | 12062364-12122247 | - | BX648696,BX649021 | INF | 3.03×10^-2^ | Dup | risk | potential | - | Wu et al(2018) |
| CNV | - | 3 | 76005745-76061858 | - | intergenic | INF | 3.03×10^-2^ | Dup | risk | potential | - | Wu et al(2018) |
| CNV | - | 10 | 6717773-6783787 | - | intergenic | 4.89 | 3.03×10^-2^ | Dup | risk | potential | - | Wu et al(2018) |
| CNV | - | 1 | 149608041-149614444 | - | SELENBP1 | 4.89 | 3.03×10^-2^ | Dup | risk | potential | - | Wu et al(2018) |
| CNV | - | 7 | 61631605-61631605 | - | intergenic | 3.74 | 3.03×10^-2^ | Dup | risk | potential | - | Wu et al(2018) |
| CNV | - | 19 | 32620453-32675491 | - | intergenic | 0.15 | 3.03×10^-2^ | Del | protective | potential | - | Wu et al(2018) |
| CNV | - | 11 | 50432844-50586426 | - | intergenic | 0.25 | 2.93×10^-2^ | Del | protective | potential | - | Wu et al(2018) |
| CNV | - | 12 | 36785447-36801139 | - | intergenic | 0.22 | 2.71×10^-5^ | Del | protective | potential | - | Wu et al(2018) |
| CNV | - | 19 | 24228244-24354405 | - | intergenic | 0.27 | 2.54×10**^-16^** | Del | protective | identified | - | Wu et al(2018) |
| CNV | - | 9 | 11941222-12175185 | - | intergenic | 0.28 | 2.44×10^-2^ | Del | protective | potential | - | Wu et al(2018) |
| CNV | - | 5 | 99455964-99486169 | - | intergenic | 0.04 | 2.44×10^-2^ | Dup | protective | potential | - | Wu et al(2018) |
| CNV | - | 8 | 145079175-145131413 | - | PARP10,PLEC1 | 0.14 | 2.39×10**^-28^** | Dup | protective | identified | - | Wu et al(2018) |
| CNV | - | 8 | 114044747-114181033 | - | CSMD3 | 0.41 | 1.85×10^-2^ | Dup | protective | potential | - | Wu et al(2018) |
| CNV | - | 1 | 187833278-187886901 | - | intergenic | 0.16 | 1.27×10^-2^ | Dup | protective | potential | - | Wu et al(2018) |
| CNV | - | 4 | 162057165-162075793 | - | intergenic | 0.58 | 1.27×10^-2^ | Dup | protective | potential | - | Wu et al(2018) |
| CNV | - | 12 | 22509872-22573948 | - | KIAA0528 | 0 | 1.26×10^-2^ | Dup | protective | potential | - | Wu et al(2018) |
| CNV | - | 19 | 21936987-21995054 | - | ZFS-3,ZNF208 | 0 | 1.19×10^-2^ | Dup | protective | potential | - | Wu et al(2018) |
| CNV | - | 4 | 145164558-145169643 | - | GYPA | 0.21 | 1.12×10^-2^ | Dup | protective | potential | - | Wu et al(2018) |
| CNV | - | 7 | 53164643-53216869 | - | intergenic | 0 | 1.08×10^-2^ | Dup | protective | potential | - | Wu et al(2018) |

**Reference**

1. Cui X, Yan H, Ou TW, Jia CS, Wang Q, Xu JJ. Genetic Variations in Inflammatory Response Genes and Their Association with the Risk of Prostate Cancer. Biomed Res Int. 2015;2015:674039. doi:10.1155/2015/674039
2. Han X, Zhang JJ, Yao N, et al. Polymorphisms in NFKB1 and NFKBIA Genes Modulate the Risk of Developing Prostate Cancer among Han Chinese. Med Sci Monit. 2015;21:1707-1715. Published 2015 Jun 12. doi:10.12659/MSM.893471
3. Li Q, Gu C, Zhu Y, et al. Two novel PRKCI polymorphisms and prostate cancer risk in an Eastern Chinese Han population. Mol Carcinog. 2015;54(8):632-641. doi:10.1002/mc.22130
4. Li XH, Xu Y, Yang K, et al. Association of THADA, FOXP4, GPRC6A/RFX6 genes and 8q24 risk alleles with prostate cancer in Northern Chinese men. J BUON. 2015;20(5):1223-1228.
5. Liu M, Miao N, Zhu Y, et al. Zhonghua Bing Li Xue Za Zhi. 2016;45(7):451-456. doi:10.3760/cma.j.issn.0529-5807.2016.07.005
6. Liu T, Gulinaer A, Shi X, et al. Gene polymorphisms in the PI3K/AKT/mTOR signaling pathway contribute to prostate cancer susceptibility in Chinese men. Oncotarget. 2017;8(37):61305-61317. Published 2017 May 22. doi:10.18632/oncotarget.18064
7. Mao X, Li J, Xu X, et al. Involvement of different mechanisms for the association of CAG repeat length polymorphism in androgen receptor gene with prostate cancer. Am J Cancer Res. 2014;4(6):886-896. Published 2014 Nov 19.
8. Long QZ, Du YF, Ding XY, et al. Replication and fine mapping for association of the C2orf43, FOXP4, GPRC6A and RFX6 genes with prostate cancer in the Chinese population. PLoS One. 2012;7(5):e37866. doi:10.1371/journal.pone.0037866
9. Wang M, Liu F, Hsing AW, et al. Replication and cumulative effects of GWAS-identified genetic variations for prostate cancer in Asians: a case-control study in the ChinaPCa consortium. Carcinogenesis. 2012;33(2):356-360. doi:10.1093/carcin/bgr279
10. Zhang LL, Sun L, Zhu XQ, et al. rs10505474 and rs7837328 at 8q24 cumulatively confer risk of prostate cancer in Northern Han Chinese. Asian Pac J Cancer Prev. 2014;15(7):3129-3132. doi:10.7314/apjcp.2014.15.7.3129
11. Zhao C, Xu H, Xu B. The rs4705342 gene mutation in the promoter region of the miR-143/145 cluster associated with the risk of prostate cancer in the Chinese Han population. Natl J Androl. 2019;8:696-702. doi:10.13263/j.cnki.nja.2019.08.004
12. Zhao L, Zheng W, Li C. Association of long-chain non-coding RNA GAS5 gene polymorphisms with prostate cancer risk and prognosis in Chinese Han population. Medicine (Baltimore). 2020;99(36):e21790. doi:10.1097/MD.0000000000021790
13. Zhang M, Wang Y, Wang C, et al. Association of Hsa-miR-23a rs3745453 variation with prostate cancer risk among Chinese Han population: A case-control study. Medicine (Baltimore). 2019;98(52):e18523. doi:10.1097/MD.0000000000018523
14. Huang WJ, Wu LJ, Min ZC, et al. Interleukin-6 -572G/C polymorphism and prostate cancer susceptibility. Genet Mol Res. 2016;15(3):10.4238/gmr.15037563. Published 2016 Sep 16. doi:10.4238/gmr.15037563
15. Wang M, Li Q, Gu C, et al. Polymorphisms in nucleotide excision repair genes and risk of primary prostate cancer in Chinese Han populations. Oncotarget. 2017;8(15):24362-24371. doi:10.18632/oncotarget.13848
16. Li Q, Gu C, Zhu Y, et al. Polymorphisms in the mTOR gene and risk of sporadic prostate cancer in an Eastern Chinese population [published correction appears in PLoS One. 2014;9(1). doi:10.1371/annotation/bc36c048-60e4-48a2-908d-6253e93df062]. PLoS One. 2013;8(8):e71968. Published 2013 Aug 5. doi:10.1371/journal.pone.0071968
17. Qu YY, Zhou SX, Zhang X, et al. Functional variants of the 5-methyltetrahydrofolate-homocysteine methyltransferase gene significantly increase susceptibility to prostate cancer: Results from an ethnic Han Chinese population. Sci Rep. 2016;6:36264. Published 2016 Nov 3. doi:10.1038/srep36264
18. Zhang Y, Li P, Xu A, et al. Influence of a single-nucleotide polymorphism of the DNA mismatch repair-related gene exonuclease-1 (rs9350) with prostate cancer risk among Chinese people. Tumour Biol. 2016;37(5):6653-6659. doi:10.1007/s13277-015-4298-x
19. Gu C, Wu J, Zhu Y, Xu H, Qin X, Zhu Y, Ye D. Genetic variations of the ADIPOQ gene and risk of prostate cancer. China Oncol. 2018;9:665-670. doi:10.19401/j.cnki.1007-3639.2018.09.004.
20. Wu JL, Zhou SX, Zhao R, et al. MTHFR c.677C>T Inhibits Cell Proliferation and Decreases Prostate Cancer Susceptibility in the Han Chinese Population in Shanghai. Sci Rep. 2016;6:36290. Published 2016 Nov 7. doi:10.1038/srep36290
21. Sun J, Lin W, Wang Q, et al. The Cell Cycle Checkpoint Gene, RAD17 rs1045051, Is Associated with Prostate Cancer Risk. Acta Med Okayama. 2021;75(4):415-421. doi:10.18926/AMO/62379
22. Tao L, Chen J, Zhou H, et al. A functional polymorphism in the CYR61 (IGFBP10) gene is associated with prostate cancer risk. Prostate Cancer Prostatic Dis. 2013;16(1):95-100. doi:10.1038/pcan.2012.41
23. Cao D, Gu C, Ye D, Dai B, Zhu Y. PCA3 rs544190G>A and prostate cancer risk in an eastern Chinese population. Int Braz J Urol. 2018;44(3):500-505. doi:10.1590/S1677-5538.IBJU.2017.0146
24. He BS, Pan YQ, Zhu CB. Zhonghua Nan Ke Xue. 2014;20(12):1077-1081.
25. Chen J, Cui X, Zhou H, et al. Functional promoter -31G/C variant of Survivin gene predict prostate cancer susceptibility among Chinese: a case control study. BMC Cancer. 2013;13:356. Published 2013 Jul 24. doi:10.1186/1471-2407-13-356
26. Chen C, Xin Z. Single-nucleotide polymorphism rs1058205 of KLK3 is associated with the risk of prostate cancer: A case-control study of Han Chinese men in Northeast China. Medicine (Baltimore). 2017;96(10):e6280. doi:10.1097/MD.0000000000006280
27. Chen Y, Xin X, Li J, et al. RTK/ERK pathway under natural selection associated with prostate cancer. PLoS One. 2013;8(11):e78254. Published 2013 Nov 4. doi:10.1371/journal.pone.0078254
28. Cui Y, Shi Y, Shen H, Fan Y, Cao W, Xie J, Shao Q. Susceptibility to prostate cancer in Han Chinese: Single nucleotide polymorphism analysis of 1,667 cases. Natl J Androl. 2012;12:1069-1074. doi:10.13263/j.cnki.nja.2012.12.005
29. Na R, Liu F, Zhang P, et al. Evaluation of reported prostate cancer risk-associated SNPs from genome-wide association studies of various racial populations in Chinese men. Prostate. 2013;73(15):1623-1635. doi:10.1002/pros.22629
30. Wu Y, Chen H, Jiang G, et al. Genome-wide Association Study (GWAS) of Germline Copy Number Variations (CNVs) Reveal Genetic Risks of Prostate Cancer in Chinese population. J Cancer. 2018;9(5):923-928. Published 2018 Feb 27. doi:10.7150/jca.22802

| **Section/topic** | **#** | **Checklist item** | **Reported on page #** |
| --- | --- | --- | --- |
| **TITLE** | | |  |
| Title | 1 | Identify the report as a systematic review, meta-analysis, or both. | 1 |
| **ABSTRACT** | | |  |
| Structured summary | 2 | Provide a structured summary including, as applicable: background; objectives; data sources; study eligibility criteria, participants, and interventions; study appraisal and synthesis methods; results; limitations; conclusions and implications of key findings; systematic review registration number. | 1 |
| **INTRODUCTION** | | |  |
| Rationale | 3 | Describe the rationale for the review in the context of what is already known. | 2 |
| Objectives | 4 | Provide an explicit statement of questions being addressed with reference to participants, interventions, comparisons, outcomes, and study design (PICOS). | 2 |
| **METHODS** | | |  |
| Protocol and registration | 5 | Indicate if a review protocol exists, if and where it can be accessed (e.g., Web address), and, if available, provide registration information including registration number. | NA |
| Eligibility criteria | 6 | Specify study characteristics (e.g., PICOS, length of follow up) and report characteristics (e.g., years considered, language, publication status) used as criteria for eligibility, giving rationale. | 3 |
| Information sources | 7 | Describe all information sources (e.g., databases with dates of coverage, contact with study authors to identify additional studies) in the search and date last searched. | 3 |
| Search | 8 | Present full electronic search strategy for at least one database, including any limits used, such that it could be repeated. | 3 |
| Study selection | 9 | State the process for selecting studies (i.e., screening, eligibility, included in systematic review, and, if applicable, included in the meta analysis). | 3 |
| Data collection process | 10 | Describe method of data extraction from reports (e.g., piloted forms, independently, in duplicate) and any processes for obtaining and confirming data from investigators. | 3 |
| Data items | 11 | List and define all variables for which data were sought (e.g., PICOS, funding sources) and any assumptions and simplifications made. | 3-4 |
| Risk of bias in individual studies | 12 | Describe methods used for assessing risk of bias of individual studies (including specification of whether this was done at the study or outcome level), and how this information is to be used in any data synthesis. | 4 |
| Summary measures | 13 | State the principal summary measures (e.g., risk ratio, difference in means). | NA |
| Synthesis of results | 14 | Describe the methods of handling data and combining results of studies, if done, including measures of consistency (e.g., I^2^ for each meta analysis.  ) | NA |

Page 1 of 2

| **Section/topic** | **#** | **Checklist item** | **Reported on page #** |
| --- | --- | --- | --- |
| Risk of bias across studies | 15 | Specify any assessment of risk of bias that may affect the cumulative evidence (e.g., publication bias, selective reporting within studies). | NA |
| Additional analyses | 16 | Describe methods of additional analyses (e.g., sensitivity or subgroup analyses, meta-regression), if done, indicating which were pre specified. | NA |
| **RESULTS** | | |  |
| Study selection | 17 | Give numbers of studies screened, assessed for eligibility, and included in the review, with reasons for exclusions at each stage, ideally with a flow diagram. | 4, 23 |
| Study characteristics | 18 | For each study, present characteristics for which data were extracted (e.g., study size, PICOS, follow-up period) and provide the citations. | 24-29 |
| Risk of bias within studies | 19 | Present data on risk of bias of each study and, if available, any outcome level assessment (see item 12). | 24-29 |
| Results of individual studies | 20 | For all outcomes considered (benefits or harms), present, for each study: (a) simple summary data for each intervention group (b) effect estimates and confidence intervals, ideally with a forest plot. | 24-29 |
| Synthesis of results | 21 | Present results of each meta-analysis done, including confidence intervals and measures of consistency. | NA |
| Risk of bias across studies | 22 | Present results of any assessment of risk of bias across studies (see Item 15). | NA |
| Additional analysis | 23 | Give results of additional analyses, if done (e.g., sensitivity or subgroup analyses, meta-regression [see Item 16]). | NA |
| **DISCUSSION** | | |  |
| Summary of evidence | 24 | Summarize the main findings including the strength of evidence for each main outcome; consider their relevance to key groups (e.g., healthcare providers, users, and policy makers). | 9-12 |
| Limitations | 25 | Discuss limitations at study and outcome level (e.g., risk of bias), and at review-level (e.g., incomplete retrieval of identified research, reporting bias). | 11-12 |
| Conclusions | 26 | Provide a general interpretation of the results in the context of other evidence, and implications for future research. | 12 |
| **FUNDING** | | |  |
| Funding | 27 | Describe sources of funding for the systematic review and other support (e.g., supply of data); role of funders for the systematic review. | 12 |

*From:* Moher D, Liberati A, Tetzlaff J, Altman DG, The PRISMA Group (2009). Preferred Reporting Items for Systematic Reviews and Meta-Analyses: The PRISMA Statement. PLoS Med 6(6): e1000097. doi:10.1371/journal.pmed1000097

**Supplementary Table 6.** **PRISMA 2009 Checklist.**
